# Neuromuscular Defects in a *Drosophila* Model of the Congenital Disorder of Glycosylation SLC35A2-CDG

**DOI:** 10.3390/biom15091256

**Published:** 2025-08-29

**Authors:** Kazuyoshi Itoh, Masaki Kurogochi, Tadashi Kaname, Jun-ichi Furukawa, Shoko Nishihara

**Affiliations:** 1 Glycan and Life Systems Integration Center (GaLSIC), Soka University, Tokyo 192-8577, Japan; kazuyoshi@soka.ac.jp; 2 Institute for Glyco-Core Research (iGCORE), Nagoya University, Nagoya 464-8601, Japan; kurogochi.masaki.d2@f.mail.nagoya-u.ac.jp (M.K.); furukawa.junichi.n0@f.mail.nagoya-u.ac.jp (J.-i.F.); 3 Department of Genome Medicine, National Center for Child Health and Development, Tokyo 157-8535, Japan; kaname-t@ncchd.go.jp; 4 Department of Orthopaedic Surgery, Faculty of Medicine and Graduate School of Medicine, Hokkaido University, Sapporo 060-8638, Japan; 5 Department of Biosciences, Graduate School of Science and Engineering, Soka University, Tokyo 192-8577, Japan

**Keywords:** SLC35A2-CDG, Ugalt, mucin-type *O*-glycan, T antigen, *Drosophila*, neuromuscular junction, muscle, basement membrane

## Abstract

SLC35A2-CDG is a congenital disorder of glycosylation caused by mutations in the *SLC35A2* gene encoding a Golgi-localized UDP-galactose transporter. This transporter plays an essential role in glycan synthesis by transporting UDP-galactose from the cytoplasm into the Golgi lumen. Its dysfunction leads to impaired galactose-containing glycans and various neurological symptoms, although the underlying mechanisms remain largely unknown. We identified a novel SLC35A2-CDG patient carrying a pathogenic variant (c.617_620del, p.(Gln206ArgfsTer45)) who exhibited neurological abnormalities including bilateral ventriculomegaly. To investigate the disease mechanism, we established the first *Drosophila* model of SLC35A2-CDG. Knockout of *Ugalt*, the fly ortholog of *SLC35A2*, resulted in embryonic lethality, indicating its essential role. Knockdown of *Ugalt* reduced mucin-type *O*-glycans on muscles and neuromuscular junctions (NMJs), without affecting *N*-glycans. *Ugalt* knockdown larvae exhibited mislocalized NMJ boutons accompanied by a deficiency in basement membrane components on muscles. This phenotype resembles that of mutants of *dC1GalT1* and *dGlcAT-P*, both involved in mucin-type *O*-glycosylation. Genetic interaction between *Ugalt* and *dC1GalT1* was confirmed through double knockdown and double heterozygous analyses. Given that *Drosophila* NMJs are widely used as a model for mammalian central synapses, our findings suggest that Ugalt regulates NMJ architecture via mucin-type *O*-glycosylation and provide insights into the molecular basis of neurological abnormalities in SLC35A2-CDG.

## 1. Introduction

Glycosylation, one of the most common post-translational modifications, is essential for many biological processes including protein folding, protein–ligand interactions, signal transduction, cell–cell interactions, and cell–extracellular matrix interactions. Deficiencies in glycosylation pathways cause congenital disorders of glycosylation (CDGs), a growing group of rare inherited metabolic disorders that include more than 160 diseases to date [1]. Patients with a CDG display extensive symptoms that manifest from birth because defective glycosylation causes the dysfunction of glycoproteins and glycolipids, impacting multiple organs [2]. In most CDGs, patients exhibit symptoms associated with neurological and developmental disabilities [3].

Solute carrier family 35 member A2 (SLC35A2)-CDG is an X-linked CDG caused by mutations in the *SLC35A2* gene encoding a nucleotide-sugar transporter, namely, uridine diphosphate-galactose (UDP-Gal) transporter. SLC35A2 is a multi-transmembrane protein localized to the endoplasmic reticulum (ER) and Golgi apparatus, and transports UDP-Gal, a donor substrate for glycosyltransferases, from the cytoplasm into their lumen [4,5,6]. Thus, SLC35A2 is essential for the biosynthesis of Gal-containing glycans. Patients affected with SLC35A2-CDG display hypogalactosylation of *N*-glycans and severe neurological symptoms such as epilepsy, intellectual disability, cerebral atrophy, and hypotonia [7,8,9,10,11,12,13,14]. However, the pathological mechanisms underlying these symptoms remain largely unknown.

In humans, somatic variants of *SLC35A2* are associated with mild malformation of cortical development with oligodendroglial hyperplasia in epilepsy (MOGHE) [15,16]. Recent studies have revealed that mice with mosaic knockout or conditional knockout of *Slc35a2* in cortical neurons exhibit features similar to human MOGHE phenotypes, including seizures and impaired neural migration [17,18,19]. In contrast, a model of *SLC35A2*-related CDGs in *Drosophila* has not been reported so far. In *Drosophila*, the ortholog of *SLC35A2* (DIOPT score, 13/16) is *Ugalt* (also known as *Csat*), which encodes a Golgi-localized multi-transmembrane UDP-Gal transporter [6,20,21]. Both human SLC35A2 and Ugalt are known to transport UDP-*N*-acetylgalactosamine (GalNAc), in addition to UDP-Gal [6]. We previously reported that whole-body knockdown (KD) of *Ugalt* leads to larval lethality, suggesting that Ugalt is essential for development; in addition, KD of *Ugalt* in histoblasts causes abdominal depigmentation in adult flies, although the underlying mechanism remains unknown [22].

SLC35A2 interacts with several glycosyltransferases involved in the synthesis of *N*-glycans [23,24,25,26], glycosaminoglycans [27], and glycolipids [28]. A recent study revealed that SLC35A2 also associates with core 1 β1,3-galactosyltransferase 1 (C1GalT1, also known as T-synthase) and Cosmc, a C1GalT1-specific chaperone, both of which are essential for mucin-type *O*-glycosylation [29]. Notably, SLC35A2-deficient cells cannot synthesize mucin-type *O*-glycans [30,31]. Collectively, these data indicate that SLC35A2 forms heterologous complexes with multiple glycosyltransferases to achieve galactosylation.

**Figure 1:**
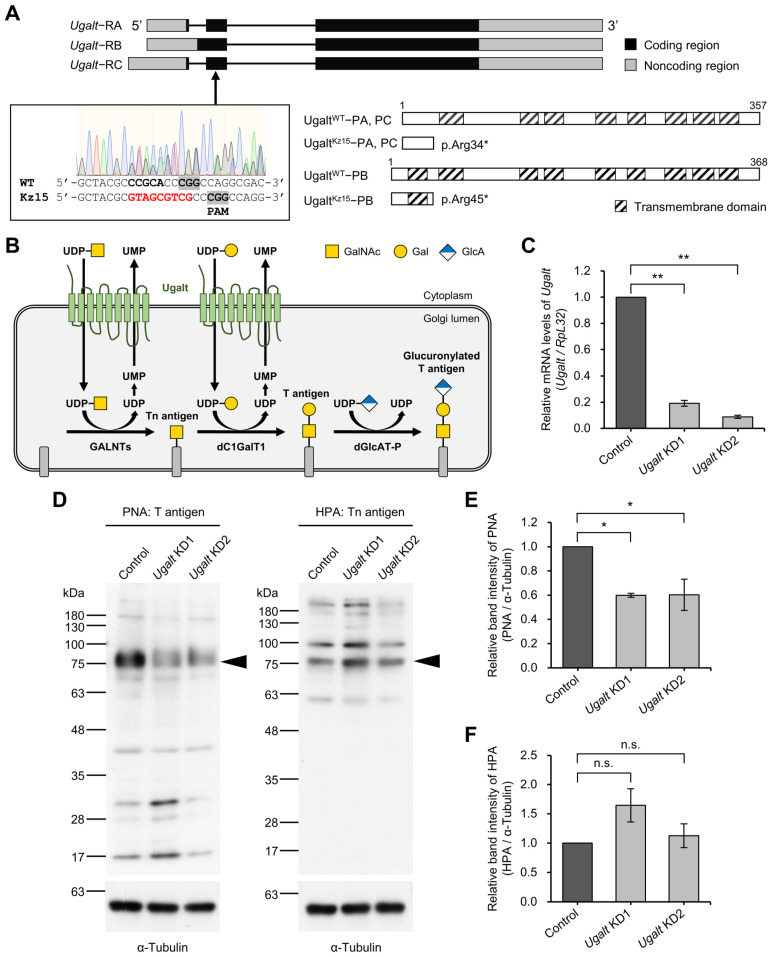
Requirement of Ugalt for mucin-type *O*-glycosylation. (**A**) Exon–intron structure of three splicing variants of *Ugalt* (upper), DNA sequence analysis of *Ugalt^Kz15^* heterozygous (*Ugalt^Kz15^/+*) mutants generated by CRISPR-Cas9 (lower left), and expected translational products from wild-type *Ugalt* (*Ugalt^WT^*) and *Ugalt^Kz15^* alleles (lower right). In the *Ugalt^Kz15^* allele, 9 bases (GTAGCGTCG) were inserted into a site 3 bases upstream of the PAM sequence in place of 5 bases (CCGCA) in *Ugalt^WT^*. The *Ugalt^WT^* allele produces two WT proteins, Ugalt^WT^-PA/PC (357 aa) and Ugalt^WT^-PB (368 aa), from *Ugalt*-RA/RC and *Ugalt*-RB, respectively. The *Ugalt^Kz15^* allele produces two truncated proteins, Ugalt^Kz15^-PA/PC (p.Arg34*) and Ugalt^Kz15^-PB (p.Arg45*), due to the nonsense mutation. (**B**) Biosynthetic pathway of mucin-type *O*-glycans in *Drosophila*. The multiple transmembrane protein Ugalt is localized to the Golgi membrane, where it transports nucleotide-sugars including uridine diphosphate-*N*-acetylgalactosamine (UDP-GalNAc) and UDP-galactose (UDP-Gal) from the cytoplasm to the Golgi lumen in exchange for uridine monophosphate (UMP) derived from UDP, a by-product of the glycosyltransferase reaction. UDP-GalNAc is a donor substrate for polypeptide *N*-acetylgalactosaminyl-transferases (GALNTs), which transfer GalNAc to the serine or threonine residue of a core-protein via an α1-linkage to synthesize Tn antigen. UDP-Gal is a donor substrate for *Drosophila* core 1 β1,3-galactosyltransferase 1 (dC1GalT1), which transfers Gal to GalNAc of Tn antigen in a β1,3-linkage to synthesize T antigen. *Drosophila* β1,3-glucuronyltransferase-P (dGlcAT-P) subsequently transfers glucuronic acid (GlcA) to the Gal residue of T antigen in a β1,3-linkage to synthesize glucuronylated T antigen. (**C**) Relative mRNA levels of *Ugalt* in *Ugalt* knockdown 1 (KD1) and *Ugalt* KD2 larvae using *Act5C-Gal4*. mRNA levels were normalized to *RpL32* mRNA, and that of *Ugalt* in the control (*Act5C-Gal4/+*) larvae was set as 1.0. Data are the mean ± standard error for each genotype (*n* = 3). (**D**) PNA (left) and HPA (right) lectin blot analyses of proteins extracted from the body walls of control, KD1, and KD2 larvae. The α-tubulin internal control is shown below. A representative example of three experiments is shown. Original lectin blot and Western blot images can be found in Figure S4. (**E**,**F**) Relative band intensity of PNA (**E**) and HPA (**F**) normalized to the band intensity of α-tubulin. The intensities of bands indicated by arrowheads in (**D**) were measured. Data are the mean ± standard error for each genotype (*n* = 3). In (**C**,**E**,**F**), statistical significance was assessed by Dunnett test: * *p* < 0.05; ** *p* < 0.01, n.s., not significant.

Mucin-type *O*-glycans are evolutionarily conserved across species. They are characterized by the attachment of GalNAc in an α1-linkage to the serine (Ser) or threonine (Thr) residue of a core-protein by multiple polypeptide *N*-acetylgalactosaminyl-transferases (GALNTs) [32,33]. The resulting terminal GalNAc glycan structure is called Tn antigen (GalNAcα1-Ser/Thr; Figure 1B). C1GalT1 subsequently transfers Gal to the GalNAc residue in a β1,3-linkage to synthesize T antigen (Galβ1-3GalNAcα1-Ser/Thr), also known as core 1 structure, which is the most common structure among mucin-type *O*-glycans. Mammalian C1GalT1 is inactive without its chaperone Cosmc [34]; however, *Drosophila* C1GalT1 (dC1GalT1) does not require a molecular chaperone and an equivalent protein to Cosmc has not been identified [35]. After T antigen synthesis, sialylated T antigen (Siaα2-3Galβ1-3GalNAcα1-Ser/Thr) is synthesized by ST3 β-galactoside α2,3-sialyltransferase 1 in mammals [32], whereas glucuronylated T antigen (GlcAβ1-3Galβ1-3GalNAcα1-Ser/Thr) is synthesized by β1,3-glucuronyltransferase-P (dGlcAT-P) in *Drosophila* (Figure 1B) [36,37,38,39,40,41]. These two trisaccharides are thought to have similar physiological functions because their terminal moieties, sialic acid and glucuronic acid, are both negatively charged monosaccharides.

Unusual expression of mucin-type *O*-glycans due to the loss of C1GALT1 activity is associated with several human disorders, including Tn syndrome [42,43], IgA nephropathy [44,45], and various cancers [46,47,48]. In mice, loss of either *C1galt1* or *Cosmc* leads to abnormalities in blood vessels [49,50], blood cells [50,51], intestines [52,53], kidneys [54,55], and brain [56]. In *Drosophila*, defective synthesis of T antigen also results in various phenotypes, including impaired tissue invasion of embryonic hemocytes [57], reduced numbers of larval circulating hemocytes [58], excessive differentiation of prohemocytes in larval lymph glands [59], and extension of larval ventral nerve cord [60,61]. Thus, mucin-type core 1 glycans play crucial roles in various tissues across species. In addition, we previously found that *dC1GalT1* and *dGlcAT-P* mutants exhibit several defects in larval neuromuscular junctions (NMJs), including mislocalization of boutons, reduced numbers of branches, and reduced lengths of postsynaptic densities, as well as partial loss of muscle basement membrane (BM) components due to the mislocalized boutons [40,41,62]. Therefore, glucuronylated core 1 glycans have vital functions in *Drosophila* NMJs, which in turn have been established as an excellent genetic model of glutamatergic synapses in the mammalian brain [63].

In this study, we surveyed our in-house cohort of 900 patients with rare and undiagnosed diseases and identified a male infant carrying a severe pathogenic variant of *SLC35A2* (c.617_620del, p.(Gln206ArgfsTer45)) who exhibited neurological abnormalities including bilateral ventricular enlargement. To clarify the causal relationship between defective glycosylation and neurological defects in SLC35A2-CDG, we generated a model in *Drosophila*. Knockout of *Ugalt* resulted in embryonic lethality, suggesting that Ugalt is essential for viability. We found that Ugalt is required for the synthesis of mucin-type *O*-glycans expressed on NMJs and muscle surfaces, and its loss leads to morphological defects in NMJs and partial loss of BM components on muscles, similar to the defects observed in *dC1GalT1* and *dGlcAT-P* mutants [40,41,62]. We further revealed that there is a genetic interaction between *Ugalt* and *dC1GalT1*. Collectively, our data demonstrate that Ugalt contributes to proper bouton localization and normal BM formation by providing the donor substrate essential for mucin-type *O*-glycosylation. Considering the significance of *Drosophila* NMJs for investigating mammalian central synapses, our *Drosophila* model of SLC35A2-CDG offers vital insights into the cellular and molecular mechanisms underlying the neurological abnormalities observed in SLC35A2-CDG.

## 2. Materials and Methods

### 2.1. Survey of Pathogenic Variants of SLC35A2 in Patients with Undiagnosed Disease

We screened our in-house database of 900 patients with rare and undiagnosed diseases [64,65] to identify individuals carrying pathogenic variants of *SLC35A2*. All whole-exome sequencing analyses were performed after obtaining written informed consent from the patients or their legal guardians. The in-house database studies were approved by the ethical committee of the National Center for Child Health and Development (No. 2020-326).

### 2.2. Fly Stocks and Mutant Generation

Fly stocks were raised at 25 °C using a standard cornmeal-yeast-glucose diet. The following strains were used: Canton-S (kindly provided by Dr. D. Yamamoto); *Act5C-Gal4* and *dC1GalT1^2.1^* (both from Bloomington *Drosophila* Stock Center); *UAS-Ugalt RNAi* (*10149GD*) (from Vienna *Drosophila* Resource Center); and *UAS-Ugalt RNAi* (*2675R-1*) (from Fly Stocks of the National Institute of Genetics). The crosses for the KD experiments were performed at 29 °C to maximize KD efficiency. *Ugalt* KD1 and *Ugalt* KD2 flies were produced using *UAS-Ugalt RNAi* (*10149GD*) and *UAS-Ugalt RNAi* (*2675R-1*), respectively. All other crossing experiments were performed at 25 °C. *Ugalt^Kz15^* flies were generated by the CRISPR-Cas9 method, as described previously [66].

### 2.3. Real-Time PCR Analysis

Quantitative analysis of mRNA levels by real-time PCR was performed as described previously [40,62]. In brief, total RNA was extracted from wandering third-instar larvae using TRI Reagent (Molecular Research Center, Cincinnati, OH, USA). First-strand cDNA was synthesized using Oligo dT primers (Thermo Fisher Scientific, Waltham, MA, USA) and Super Script II Reverse Transcriptase (Thermo Fisher Scientific). Real-time PCR was carried out using FastStart Universal SYBR Green Master (Roche, Basel, Switzerland) and a QuantStudio™ 12K Flex Real-Time PCR System (Thermo Fisher Scientific). Gene-specific primer sets are described in Supplemental Table S1.

### 2.4. Western Blot and Lectin Blot Analyses

Western blot and lectin blot analyses were performed as described previously [40,62]. In brief, proteins were extracted from body wall muscles of wandering third-instar larvae using lysis buffer (50 mM Tris-HCl, 150 mM NaCl, 1% Triton X-100 (Sigma-Aldrich, St. Louis, MO, USA), and protease inhibitor cocktail (Nacalai Tesque, Kyoto, Japan); pH 7.4). Samples were subjected to 10% SDS-PAGE (10 or 20 µg of total protein per lane), and the separated proteins were transferred to Immobilon-P membranes (Millipore, Billerica, MA, USA). For Western blot analysis, after incubation with 1% BSA for 1 h at room temperature, the membrane was incubated with rabbit anti-α-tubulin antibody (1:2500; GeneTex, Irvine, CA, USA) overnight at 4 °C, and then with horseradish peroxidase (HRP)-conjugated anti-rabbit IgG antibody (1:20,000; Cell Signaling Technology, Danvers, MA, USA) for 1 h at room temperature. For lectin blot analysis, the membrane was incubated with HRP-conjugated peanut agglutinin (PNA; 1:10,000; J-Oil Mills, Tokyo, Japan) and HRP-conjugated *Helix pomatia* agglutinin (HPA; 1:1000; EY Laboratories, San Mateo, CA, USA) overnight at 4 °C. Membranes were visualized with an ECL Prime Western blot detection reagent (Cytiva, Tokyo, Japan).

### 2.5. Preparation of Tissue Lysates for Glycan Analysis

Ten wandering third-instar larvae from each genotype were frozen using liquid nitrogen and homogenized in 200 µL of PBS. After centrifugation at 20,000× *g* for 5 min at 4 °C, the supernatant was harvested and stored at −20 °C until analysis.

### 2.6. O-Glycan Preparation by Evaporative β-elimination with Pyrazolone

Tissue lysates (protein 100 µg) were derivatized by incubation with Reagents A and B (100 µL) from the SialoCapper™-ID Kit (Shimadzu, Kyoto, Japan) for 1 h at room temperature with shaking [67]. Reagent C (100 µL) was then added and the reaction was incubated for 1 min with shaking. To remove excess condensation reagents, sialic acid linkage-specific alkylamidation (SALSA)-derivatized glycoproteins were precipitated by adding a 5-fold volume of MeCN (1 mL). After centrifugation at 14,000× *g* for 20 min at 4 °C, the pellet was resuspended in 90% MeCN (1 mL) and then centrifuged in the same manner. The resulting precipitate was dissolved in 560 mM NaOH/800 mM 1-phenyl-3-methyl-5-pyrazolone (PMP) in 48% MeOH (100 µL) and heated at 105 °C for 2 h. After the β-elimination reaction, bis-PMP-labeled Fucα(1-6)GlcNAc (GNF) was added as an external standard (400 pmol). The reaction was neutralized with HCl and extracted with chloroform/H_2_O. The aqueous phase including bis-PMP-labeled *O*-glycans was desalted by solid-phase extraction on C18 silica. The washing solution was 0.1% formic acid (100 µL × 2) and water (100 µL × 2); the eluent was 20% MeCN (100 µL) and 50% MeCN (100 µL). The eluate was dried by using a centrifugal concentrator and dissolved in water (50 µL).

### 2.7. N-Glycan Preparation by Glycoblotting

Tissue lysate (protein 100 µg) was incubated with 10 mM Tris(2-carboxyethyl)phosphine in 100 mM NH_4_HCO_3_ (final concentration) at room temperature for 60 min; the mixture was then alkylated by incubation with 22 mM iodoacetamide (final concentration) at room temperature for 30 min in the dark. The proteins were digested by adding trypsin (50 µg) in 50 mm NH_4_HCO_3_ at 37 °C for 2.5 h; the enzyme was then inactivated by heating at 90 °C for 15 min. After cooling, the *N*-glycans were released from the tryptic glycopeptides by incubation with PNGase F Prime (N-zyme Scientific, Doylestown, PA, USA) (334 U) at 37 °C for 6 h. For quantitative analysis, disialyloctasaccharide (A2GN1; Tokyo Chemical Industry, Tokyo, Japan) (40 pmol) was added to the reaction solution.

Glycoblotting of the samples containing released *N*-glycans was performed as previously described [68]. Released *N*-glycans were derivatized via the aminolysis-SALSA method in the solid phase [69]. SALSA-derivatized and aminooxy-functionalized tryptophanylarginine methyl ester (aoWR)-labeled *N*-glycans were collected on BlotGlyco beads (Sumitomo Bakelite, Tokyo, Japan) and purified using a HILIC elution plate (Waters, Milford, MA, USA) to remove excess reagents.

### 2.8. Glycan Analysis by MALDI-TOF/TOF Mass Spectrometry

*O*- and *N*-glycan samples (1 µL) were mixed with 2,5-dihydroxybenzoic acid (DHB) and 3 mM NaCl matrix solutions (1 µL) and deposited onto a polished steel MALDI target and allowed to dry by evaporation. All measurements were performed on a rapiflex MALDI-TOF/TOF mass spectrometer equipped with a Smartbeam 3D Nd:YAG laser pulsed at 355 nm (Bruker Daltonics, Bremen, Germany) and controlled by FlexControl 4.2 software in accordance with general protocols. Spectra were obtained in reflectron mode with the following parameters: ion source voltage, 20 kV; pulsed ion extraction voltage, 2.57 kV; lens voltage, 11.65 kV; Reflector 1 voltage, 20.85 kV; Reflector 2 voltage, 1.085 kV; Reflector 3 voltage, 8.70 kV; and pulsed ion extraction delay, 160 ns. Relative quantitative analysis was performed by comparative analyses between the areas of the mass spectrometry (MS) signals derived from each glycan and a known amount of the internal standard (A2GN1, 40 pmol; and GNF, 400 pmol/100 µg of protein).

### 2.9. Immunostaining and Lectin Staining

Immunostaining and lectin staining were performed as described previously [40,62]. In brief, dissected wandering third-instar larvae were fixed with 4% paraformaldehyde in PBS for 20 min at room temperature. Both rabbit anti-HRP antibody (1:300; Jackson ImmunoResearch, West Grove, PA, USA) and mouse anti-Fasciclin II (Fas II) antibody (1:20; Developmental Studies Hybridoma Bank, Iowa City, IA, USA) were used to label NMJ boutons. Rabbit anti-Nidogen (Ndg) antibody (1:500; kindly provided by A. Holz) and Acti-stain 555 phalloidin (1:300; Cytoskeleton, Denver, CO, USA) were used to label the BM and muscle fibers, respectively. These antibodies and phalloidin were incubated overnight at 4 °C. Alexa Fluor-488-conjugated PNA (PNA-488; 1:50; J-Oil Mills) and rhodamine-conjugated HPA (HPA-rhodamine; 1:100; EY Laboratories) were used to label T antigen and Tn antigen, respectively, without permeabilization. These lectins and secondary antibodies, including Cy5-conjugated anti-rabbit IgG, Cy5-conjugated anti-mouse IgG, and Alexa Fluor-488-conjugated anti-rabbit IgG (all at 1:300; Thermo Fisher Scientific) were incubated for 2.5 h at room temperature. Tissue images were collected on an LSM700 confocal laser microscope (Carl Zeiss, Jena, Germany). Relative fluorescence intensities of PNA and HPA on the surface of muscles and NMJs were quantified by using ZEN 2012 software (Black Edition, Carl Zeiss). Three-dimensional images were constructed by Imaris 9.3.1 software (Bitplane, Belfast, UK).

### 2.10. Generation of the Rescue Construct

The rescue construct, *UAS-Ugalt*, was generated by the Gateway cloning technique (Thermo Fisher Scientific), as described previously [59,70]. The full-length coding sequence of *Ugalt* (GenBank: BT003169) was amplified by two-step PCR. In the first PCR, cDNA clone SD16302 (Drosophila Genomics Resource Center) was used as a template. In the second PCR, the first PCR product was used as a template. Primer sets are listed in Supplemental Table S2. The amplified fragment was subcloned into the pDONR201™ vector (Thermo Fisher Scientific). The inserted fragment was then recombined into pUAST vector to yield pUAST-*Ugalt*. Microinjection of the vector into *Drosophila* embryos and generation of transgenic flies were performed by BestGene (Chino Hills, CA, USA).

### 2.11. Statistical Analysis

Statistical evaluation of differences between the groups was carried out by using Student’s *t*-test or multiple comparison tests, including the Dunnett test, Steel test, and Steel-Dwass test, implemented in Excel (Microsoft, Redmond, WA, USA). Differences were considered to be significant at a *p*-value of less than 0.05.

## 3. Results

### 3.1. Neurological Abnormalities in a Patient with a Pathogenic Variant of the SLC35A2 Gene

From the whole-exome sequencing data of 900 patients, we identified a male infant carrying a pathogenic hemizygous variant (NM_001032289.3:c.617_620del,p.(Gln206Argfster45)) in *SLC35A2* located on the chromosome X. During the prenatal period, the infant was noted to have ventricular enlargement and multicystic renal dysplasia. He was born at 34 weeks and 4 days of gestation, with a birth weight of 1876 g. His head circumference was −1.2 standard deviations below the mean. Brain CT and MRI scans revealed bilateral ventricular enlargement. However, neonatal auditory evoked potentials were within normal limits. Abdominal ultrasonography demonstrated multicystic dysplastic kidney on the right side, without any evidence of lower ureteral dilation. By 16 days of age, no obvious seizures had been observed in him. There was no family history of any of these symptoms. His mother was a heterozygous carrier of the same variant with no clinical findings. Chromosomal analysis revealed a 46,XY karyotype.

### 3.2. Drosophila Ortholog Ugalt Is Essential for Viability

To generate a *Drosophila* model of SLC35A2-CDG, we first constructed a null allele of *Ugalt*, *Ugalt^Kz15^*, by using the CRISPR-Cas9 method [66]. Two splicing isoforms, Ugalt-PA/PC (357 amino acids; aa) and Ugalt-PB (368 aa), are produced from the wild-type *Ugalt* gene located on the X chromosome (Figure 1A; https://flybase.org/reports/FBgn0024994; accessed on 26 February 2025). In the *Ugalt^Kz15^* allele, a nonsense mutation was introduced within the coding region, resulting in the production of two truncated proteins, Ugalt^Kz15^-PA/PC (p.Arg34*) and Ugalt^Kz15^-PB (p.Arg45*), which would have no function as a nucleotide sugar transporter.

Notably, larvae of *Ugalt^Kz15^* hemizygous and homozygous mutants were not observed, suggesting that the mutation was embryonic lethal. Next, therefore, we examined whether the lethality could be rescued by ubiquitous expression of the *Ugalt* gene. We constructed *UAS-Ugalt* flies and confirmed the overexpression of *Ugalt* relative to *Act5C-Gal4/+* larvae by crossing them with *Act5C-Gal4* flies (Supplemental Figure S1A). The lethality of *Ugalt^Kz15^* hemizygous mutants was rescued by ubiquitous expression of *Ugalt* encoding Ugalt-PA/PC (Supplemental Figure S1B), showing that *Ugalt* is essential for viability.

### 3.3. Ugalt Is Required for the Synthesis of Mucin-Type O-Glycans

Given the function of Ugalt as a Golgi-localized UDP-Gal transporter (Figure 1B), its loss is likely to lead to reduced levels of Gal-containing mucin-type *O*-glycans. To examine this, we performed whole-body KD of *Ugalt* using the *Act5C-Gal4* driver because *Ugalt^Kz15^* mutants showed embryonic lethality, as described above. The relative mRNA levels of *Ugalt* in *Ugalt* KD1 and KD2 larvae were reduced to, respectively, about 20% and 10% of those in control (*Act5C-Gal4/+*) larvae (Figure 1C). Next, we estimated the expression levels of T antigen and Tn antigen in *Ugalt* KD larvae by lectin blot analyses using PNA and HPA, respectively. Similarly to the results of our previous analysis in *dC1GalT1* mutants [62], in the PNA blot for T antigen, the intensities of bands near 75 kDa were significantly decreased in the two *Ugalt* KD larvae as compared with the control (*Act5C-Gal4/+*) larvae (arrowhead in Figure 1D, left panel; Figure 1E; and Supplemental Figure S4), although the overall band intensities in the whole lane did not differ between the control and two *Ugalt* KD larvae (Figure 1D, left panel; and Supplemental Figure S2A). Therefore, this result suggests that Ugalt is required for synthesis of T antigen. In the HPA blot for Tn antigen; however, the intensities of bands were comparable between the control and two KD larvae (Figure 1D, right panel; Figure 1F; and Supplemental Figures S2B and S4). Although the levels of Tn antigen would be expected to increase due to reduced synthesis of T antigen, this result suggests that increased levels of Tn antigen are suppressed by an insufficient supply of UDP-GalNAc, a substrate essential for Tn antigen synthesis, because Ugalt transports UDP-GalNAc in addition to UDP-Gal (Figure 1B) [6].

### 3.4. Ugalt Deficiency Selectively Reduces Mucin-Type O-Glycans But Not N-Glycans

**Figure 2:**
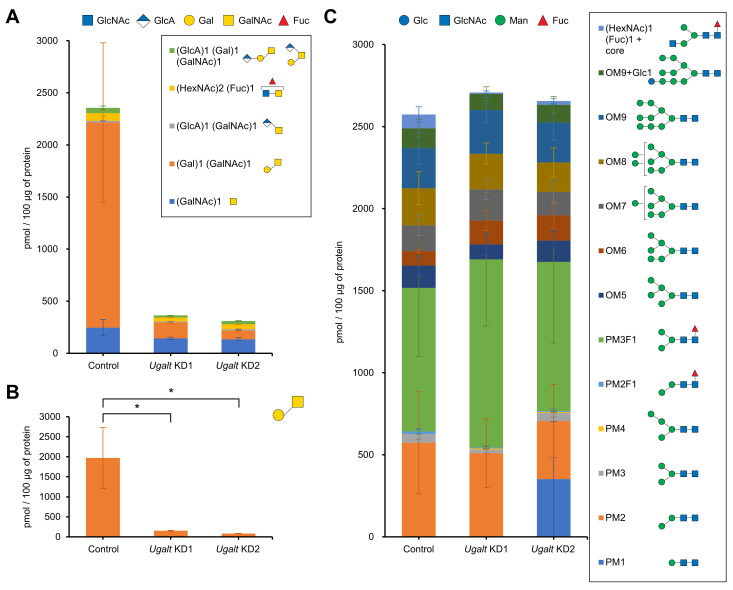
*O*-linked and *N*-linked glycan profiles of *Ugalt* knockdown larvae. (**A**) Total *O*-linked glycan profile of control (*Act5C-Gal4/+*), *Ugalt* knockdown 1 (KD1) and *Ugalt* KD2 third-instar larvae. GlcNAc, *N*-acetylglucosamine; GlcA, glucuronic acid; Gal, galactose; GalNAc, *N*-acetylgalactosamine; Fuc, fucose. (**B**) Amount of T antigen in control, *Ugalt* KD1, and *Ugalt* KD2 larvae. Statistical significance was assessed by Dunnett test: * *p* < 0.05. (**C**) Total *N*-linked glycan profile of control, *Ugalt* KD1, and *Ugalt* KD2 larvae. Glc, glucose; Man, mannose; OM, oligomannose; PM, paucimannose. In (**A**–**C**), data are the mean ± standard error for each genotype (*n* = 3).

To further investigate whether the loss of Ugalt reduces T antigen, we used MS to analyze the *O*-glycans released from glycoproteins by β-elimination. The amount of T antigen was significantly reduced in the two *Ugalt* KD third-instar larvae as compared with controls, whereas the amount of Tn antigen was comparable among the control and two *Ugalt* KD larvae (Figure 2A,B). These data clearly show that the loss of Ugalt leads to a significant decrease in T antigen levels, but does not affect Tn antigen levels, consistent with the results of the lectin blot analyses (Figure 1D–F). T antigen levels were lower in *Ugalt* KD2 larvae than in *Ugalt* KD1 larvae (Figure 2A,B). In addition, the levels of glucuronylated T antigen, including linear (GlcAβ1-3Galβ1-3GalNAc) and branched (Galβ1-3(GlcA1-4)GalNAc) forms, tended to be lower in the two *Ugalt* KD larvae as compared with controls (Figure 2A).

We also examined whether the levels of *N*-glycans are affected by the loss of Ugalt by using MS to analyze the *N*-glycans released from glycoprotein after PNGase F digestion. There was no considerable difference in the composition of *N*-glycans between the control and two *Ugalt* KD larvae (Figure 2C). A previous MS study detected galactosylated *N*-glycans as minor components in *Drosophila* embryos [71]; however, those glycan structures were not detected in the third-instar larvae of any genotype in our glycan analysis. Therefore, the loss of Ugalt hardly affects *N*-glycan composition.

Collectively, these data demonstrate that *Ugalt* deficiency reduces mucin-type *O*-glycans, including T antigen and its glucuronylated form, but has little impact on the profile of *N*-glycans.

### 3.5. Loss of Ugalt Leads to Reduced T antigens on the Surfaces of Muscles and NMJs

**Figure 3:**
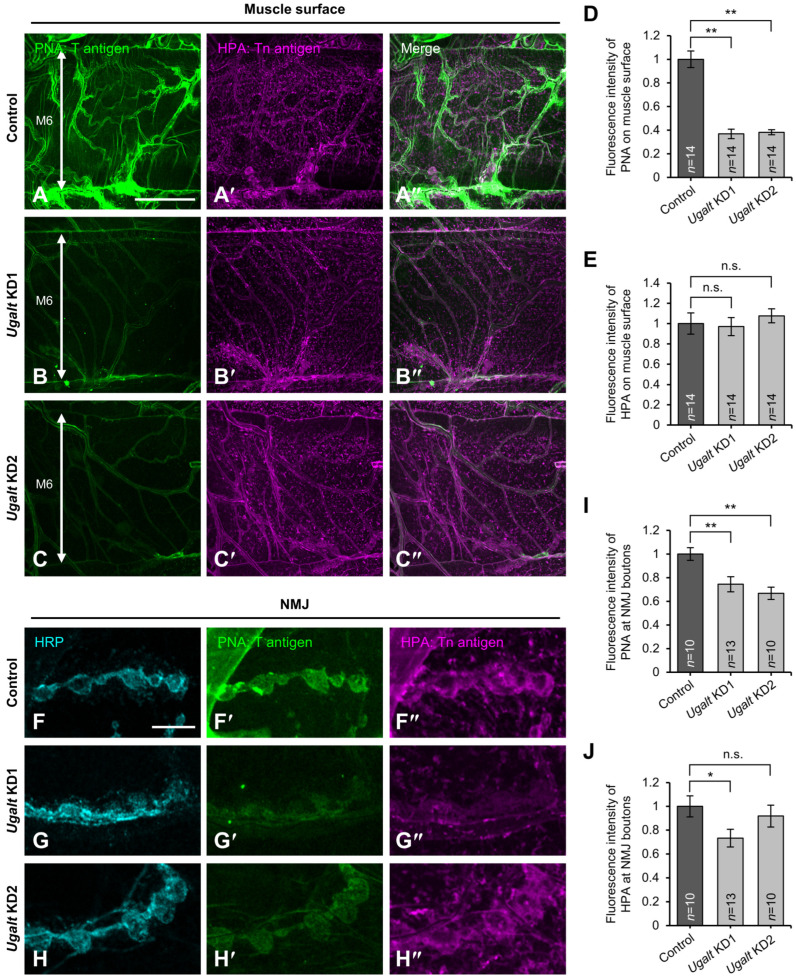
Reduced T antigen expression on the surfaces of muscles and neuromuscular junctions in *Ugalt* knockdown larvae. (**A**–**C″**) Confocal images of muscle 6 (M6) at abdominal segment 3 in control (*Act5C-Gal4/+*) (**A**–**A″**), *Ugalt* knockdown 1 (KD1) (**B**–**B″**), and *Ugalt* KD2 (**C**–**C″**) third-instar larvae. Surface sectional views are shown. Double-headed arrow indicates the width of M6. T antigen and Tn antigen were stained with PNA-488 (green) and HPA-rhodamine (magenta), respectively, without permeabilization. Scale bar: 50 µm. (**D**,**E**) Relative fluorescence intensities of PNA-488 (**D**) and HPA-rhodamine (**E**) on muscle surfaces normalized to the average of wild-type (WT) data. Data are the mean ± standard error for each genotype (*n* = 14). (**F**–**H″**) Confocal images of neuromuscular junctions (NMJs) on muscle 4 at abdominal segment 3 in control (*Act5C-Gal4/+*; **F**–**F″**), *Ugalt* KD1 (**G**–**G″**), and *Ugalt* KD2 (**H**–**H″**) third-instar larvae. NMJs were labeled with anti-HRP antibody (a presynaptic marker; cyan), PNA-488 (green), and HPA-rhodamine (magenta) without permeabilization. Scale bar: 10 µm. (**I**,**J**) Relative fluorescence intensities of PNA-488 (**I**) and HPA-rhodamine (**J**) at NMJs normalized to the average of WT data. Data are the mean ± standard error for each genotype (*n* = 10–13). In (**D**,**E**,**I**,**J**), statistical significance was assessed by Dunnett test (**D**,**I**) and Steel test (**E**,**J**): * *p* < 0.05, ** *p* < 0.01; n.s., not significant.

Next, we examined whether the levels of T antigen are decreased on muscle and NMJ surfaces in whole-body *Ugalt* KD larvae by lectin staining without permeabilization. T antigen expression was significantly decreased on the surface of both muscles (Figure 3A–D) and NMJs (Figure 3F–I) in the two *Ugalt* KD larvae as compared with control (*Act5C-Gal4/+*) larvae. In contrast, Tn antigen expression was not increased on the surface of either muscles (Figure 3A′–C″,E) or NMJs (Figure 3F″–H″,J) in the two KD larvae relative to control larvae. Consistent with our lectin blot and MS analyses, these results suggest that Ugalt is involved in the synthesis of T antigen expressed on muscle and NMJ surfaces.

### 3.6. Mislocalization of NMJ Boutons at the Muscle 6/7 Boundary in Ugalt KD Larvae

**Figure 4:**
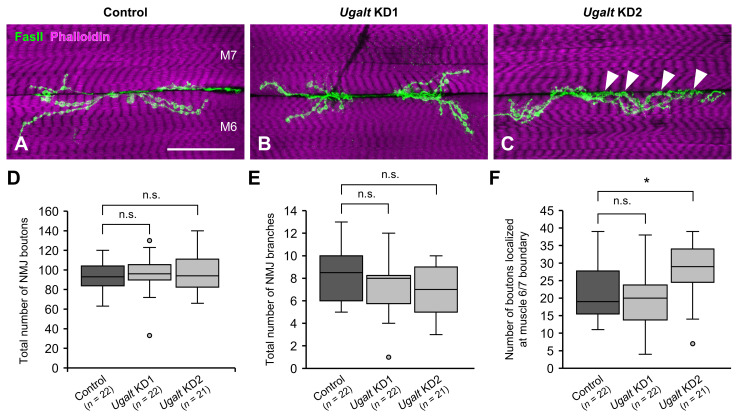
Increase in mislocalized boutons at the muscle 6/7 boundary in *Ugalt* knockdown larvae. (**A**–**C**) Confocal images of neuromuscular junctions (NMJs) on muscle 6 (M6) and M7 at abdominal segment 3 in control (*Act5C-Gal4/+*) (**A**), *Ugalt* knockdown 1 (KD1) (**B**), and *Ugalt* KD2 (**C**) third-instar larvae. NMJ boutons and muscle fibers were stained with anti-Fas II antibody (a presynaptic marker; green) and phalloidin (magenta), respectively. Maximum intensity projections are shown. Arrowheads in (**C**) indicate mislocalized boutons at the muscle 6/7 boundary. Scale bar: 50 µm. (**D**) Total number of NMJ boutons in control (*n* = 22), *Ugalt* KD1 (*n* = 22), and *Ugalt* KD2 (*n* = 21) larvae. (**E**) Total number of NMJ branches in control (*n* = 22), *Ugalt* KD1 (*n* = 22), and *Ugalt* KD2 (*n* = 21) larvae. A branch was defined as a terminal neurite containing at least two boutons. (**F**) Number of mislocalized boutons at the M6/7 boundary in control (*n* = 22), *Ugalt* KD1 (*n* = 22), and *Ugalt* KD2 (*n* = 21) larvae. In (**D**–**F**), statistical significance was assessed by Steel test (**D**,**F**) or Dunnett test (**E**): * *p* < 0.05; n.s., not significant.

In *Drosophila* larvae, the axon terminal of a motor neuron branches and establishes NMJ boutons near the boundary between abdominal muscles 6 and 7. We therefore examined whether NMJ morphologies are affected by the whole-body loss of *Ugalt*. The overall total numbers of NMJ boutons and NMJ branches were comparable between control (*Act5C-Gal4/+*) larvae and the two *Ugalt* KD larvae (Figure 4A–E). Notably, the number of NMJ boutons localized within the muscle 6/7 boundary was significantly higher in *Ugalt* KD2 but not in *Ugalt* KD1 larvae (Figure 4C, arrowheads, and Figure 4F). The observation of significant bouton mislocalization only in *Ugalt* KD2 may be attributed to the higher KD efficiency of KD2 as compared with KD1 (Figure 1C), as well as the lower T antigen levels in KD2 (Figure 2A,B). In our previous studies, this bouton mislocalization phenotype was also observed in *dC1GalT1* and *dGlcAT-P* mutants [40,41,62]. Thus, these data indicate that mislocalization of NMJ boutons due to the loss of *Ugalt* is associated with impaired synthesis of mucin-type *O*-glycans.

### 3.7. Loss of BM Components Underneath the Mislocalized NMJ Boutons in Ugalt KD Larvae

**Figure 5:**
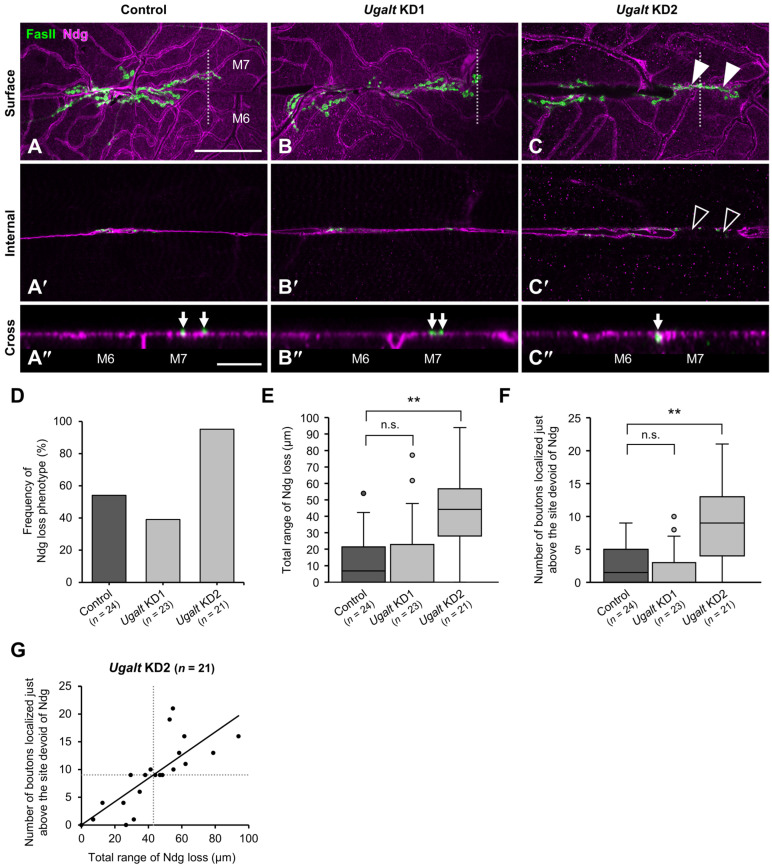
Loss of basement membrane components underneath mislocalized boutons at muscle 6/7 boundary in *Ugalt* knockdown larvae. (**A**–**C″**) Confocal images of basement membranes (BMs) and neuromuscular junctions (NMJs) on muscle 6 (M6) and M7 at abdominal segment 3 in control (*Act5C-Gal4/+*) (**A**–**A″**), *Ugalt* knockdown 1 (KD1) (**B**–**B″**), and *Ugalt* KD2 (**C**–**C″**) third-instar larvae. Shown are surface (**A**–**C**) and internal (**A′**–**C′**) sectional views of the muscles, and cross-sectional views of areas within the white dotted-lines in (**A**–**C**,**A″**–**C″**). BMs and NMJ boutons were labeled with anti-Ndg antibody (magenta) and anti-Fas II antibody as a presynaptic marker (green), respectively. White arrowheads in (**C**) and black arrowheads in (**C′**) indicate, respectively, mislocalized boutons and a partial loss of Ndg staining at the M6/7 boundary. Arrows in (**A″**–**C″**) show positions of NMJ boutons. Scale bars: 50 µm (**A**–**C**,**A′**–**C′**) and 10 µm (**A″**–**C″**). (**D**) Frequency of Ndg loss phenotype in control (*n* = 24), *Ugalt* KD1 (*n* = 23), and *Ugalt* KD2 (*n* = 21) larvae. (**E**) Total range of Ndg loss at the M6/7 boundary in control (*n* = 24), *Ugalt* KD1 (*n* = 23), and *Ugalt* KD2 (*n* = 21) larvae. (**F**) Number of boutons localized just above the site devoid of Ndg at the M6/7 boundary in control (*n* = 24), *Ugalt* KD1 (*n* = 23), and *Ugalt* KD2 (*n* = 21) larvae. In (**E**,**F**), statistical significance was assessed by Steel test: ** *p* < 0.01; n.s., not significant. (**G**) Correlation between the total range of Ndg loss and the number of boutons localized just above the site devoid of Ndg at the M6/7 boundary in *Ugalt* KD2 (*n* = 21) larvae. Pearson’s correlation coefficient *r* was 0.79. Unbroken line indicates the straight line fit; dotted lines indicate the average values.

We previously reported that BM components, including collagen IV and Ndg, were missing just beneath the mislocalized NMJ boutons at the muscle 6/7 boundary in *dC1GalT1* and *dGlcAT-P* mutants [40,41,62]. To examine whether similar defects also occur in larvae with whole-body KD of *Ugalt*, we labeled and observed the localization of Ndg, a glycoprotein that binds to collagen IV and laminin. In surface sectional views of muscles 6 and 7, Ndg was expressed on the two muscle surfaces in both control (*Act5C-Gal4/+*) and *Ugalt* KD larvae (Figure 5A–C), and the expression levels of Ndg on the muscle surfaces were comparable between the control and *Ugalt* KD larvae. In internal sectional views, Ndg was localized at the muscle 6/7 boundary in control larvae (Figure 5A′) but partially lost at the muscle 6/7 boundary (Figure 5C′, black arrowheads) just below the mislocalized boutons (Figure 5C, white arrowheads) in *Ugalt* KD2 larvae, although *Ugalt* KD1 larvae hardly showed this phenotype (Figure 5B′). In cross-sectional views, the two muscles were individually covered with separate BMs in control larvae (Figure 5A″). In *Ugalt* KD2 larvae; however, the two muscles were covered with a continuous single BM, and a bouton was mislocalized at the muscle 6/7 boundary (Figure 5C″, arrow); these abnormalities were rarely observed in *Ugalt* KD1 larvae (Figure 5B″). Both the frequency of the Ndg loss phenotype and the total range (in μm) of Ndg loss at muscle 6/7 boundary were significantly increased in *Ugalt* KD2 but not in *Ugalt* KD1 larvae as compared with control larvae (Figure 5D,E).

The number of NMJ boutons localized just above the site of missing Ndg at the muscle 6/7 boundary was also significantly increased in *Ugalt* KD2 but not in *Ugalt* KD1 larvae relative to control larvae (Figure 5F). Moreover, there was a strong correlation between the total range of missing Ndg and the number of NMJ boutons just above the site of missing Ndg at the muscle 6/7 boundary in *Ugalt* KD2 larvae (Figure 5G), suggesting that the NMJ boutons were preferentially localized at this site due to the loss of *Ugalt*.

**Figure 6:**
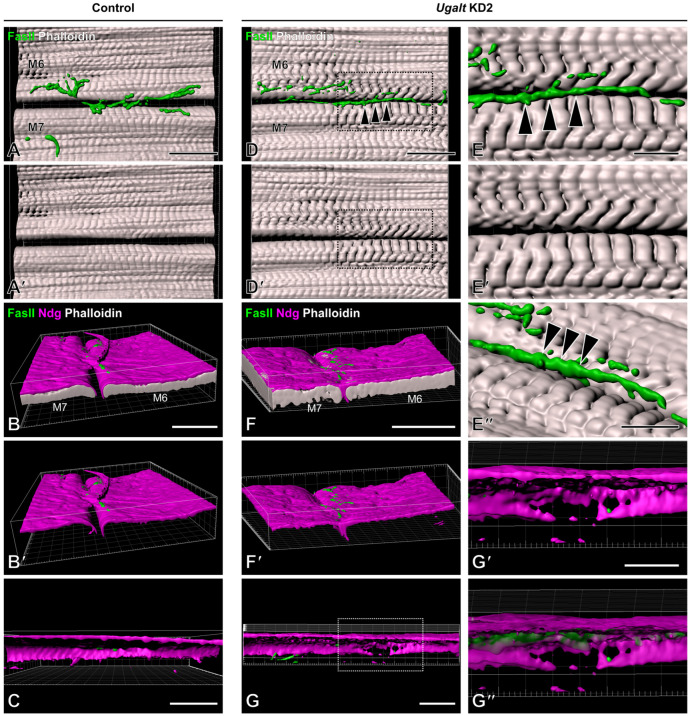
Three-dimensional view of mislocalized boutons and BM component loss in *Ugalt* knockdown larvae. Surface rendering models of three-dimensional confocal images of neuromuscular junctions (NMJs), muscle fibers, and basement membranes (BMs) in control (*Act5C-Gal4/+*) (**A**–**C**) and *Ugalt* knockdown 2 (**D**–**G″**) larvae. NMJ boutons, muscle fibers, and BMs were labeled with anti-Fas II antibody (green), phalloidin (gray), and anti-Ndg antibody (magenta), respectively. High-magnification views of the boxed area in (**D**,**D′**) are presented in (**E**,**E′**), respectively. Arrowheads in (**D**,**E**,**E″**) indicate mislocalized boutons. BMs formed between muscle 6 (M6) and M7 are shown in (**C**,**G**). High-magnification views of the boxed area in (**G**) are presented in (**G′**,**G″**). Ndg was partially lost just beneath the mislocalized boutons at the M6/7 boundary (**G**–**G″**). Scale bars: 50 µm (**A**–**D′**,**F**–**G**) and 20 µm (**E**–**E″**,**G′**,**G″**).

To visualize further the positional relationship between the mislocalized boutons and the region of missing Ndg, we constructed three-dimensional images. Although there was a cleft between muscles 6 and 7 in control larvae (Figure 6A,A′), the two muscles were close to each other just beneath the mislocalized boutons in the *Ugalt* KD2 larvae (Figure 6D–E″, arrowheads). Whereas BMs were seamlessly formed at the muscle 6/7 boundary in control larvae (Figure 6B,C and Supplemental Video S1), they were partially lost just beneath the mislocalized boutons at the boundary in *Ugalt* KD2 larvae (Figure 6F–G″ and Supplemental Video S2). These phenotypes were previously observed in *dC1GalT1* and *dGlcAT-P* mutants [40,41,62]; both the mislocalization of NMJ boutons and the loss of BM components in *Ugalt* KD larvae seem likely to result from defective synthesis of mucin-type *O*-glycans, including T antigen and glucuronylated T antigen.

### 3.8. Ugalt Genetically Interacts with dC1GalT1

**Figure 7:**
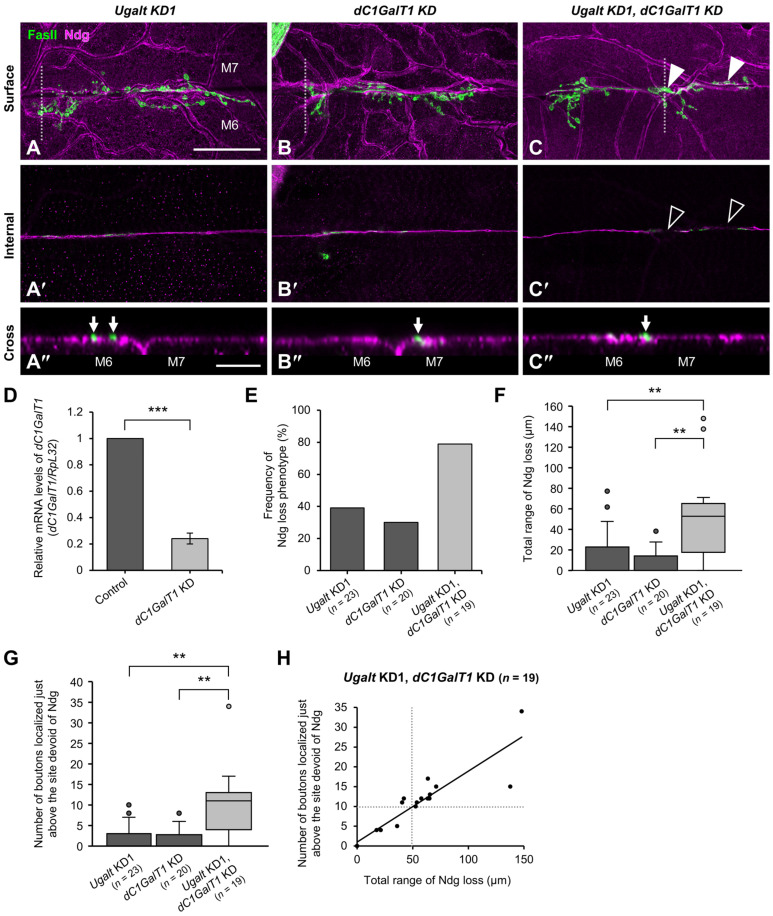
Genetic interaction between *Ugalt* and *dC1GalT1*. (**A**–**C″**) Confocal images of basement membranes (BMs) and neuromuscular junctions (NMJs) on muscle 6 (M6) and M7 at abdominal segment 3 in *Ugalt* knockdown 1 (KD1) (**A**–**A″**), *dC1GalT1* KD (**B**–**B″**), and *Ugalt* and *dC1GalT1* double KD (**C**–**C″**) third-instar larvae. Shown are surface (**A**–**C**) and internal (**A′**–**C′**) sectional views of the muscles, and cross-sectional views of areas within the white dotted-lines in (**A**–**C**,**A″**–**C″**). BMs and NMJ boutons were labeled with anti-Ndg antibody (magenta) and anti-Fas II antibody (green), respectively. White arrowheads in (**C**) and black arrowheads in (**C′**) indicate, respectively, mislocalized boutons and partial loss of Ndg staining at the M6/7 boundary. Arrows in (**A″**–**C″**) indicate NMJ boutons. Scale bars: 50 µm (**A**–**C**,**A′**–**C′**) and 10 µm (**A″**–**C″**). (**D**) Relative mRNA levels of *dC1GalT1* in *dC1GalT1* KD larvae using *Act5C-Gal4*. The mRNA level of *dC1GalT1* in the control (*Act5C-Gal4/+*) larvae was set as 1.0. Data are the mean ± standard error for each genotype (*n* = 3). (**E**) Frequency of Ndg loss phenotype in *Ugalt* KD1 (*n* = 23), *dC1GalT1* KD (*n* = 20), and *Ugalt* and *dC1GalT1* double KD (*n* = 19) larvae. (**F**) Total range of Ndg loss at the M6/7 boundary in *Ugalt* KD1 (*n* = 23), *dC1GalT1* KD (*n* = 20), and *Ugalt* and *dC1GalT1* double KD (*n* = 19) larvae. (**G**) Number of boutons localized just above the site devoid of Ndg at the M6/7 boundary in *Ugalt* KD1 (*n* = 23), *dC1GalT1* KD (*n* = 20), and *Ugalt* and *dC1GalT1* double KD (*n* = 19) larvae. In (**D**,**F**,**G**), statistical significance was assessed by Student’s *t*-test (**D**) or Steel-Dwass test (**F**,**G**): ** *p* < 0.01, *** *p* < 0.001. (**H**) Correlation between the total range of Ndg loss and the number of boutons localized just above the site devoid of Ndg at the M6/7 boundary in *Ugalt* and *dC1GalT1* double KD (*n* = 19) larvae. Pearson’s correlation coefficient *r* was 0.91. Unbroken line indicates the straight line fit; dotted lines indicate the average values.

To confirm whether both the mislocalization of boutons and the loss of BM components in *Ugalt* KD larvae are caused by an impairment of mucin-type *O*-glycosylation, we performed whole-body double KD of *Ugalt* and *dC1GalT1*. Although bouton mislocalization accompanied by partial loss of Ndg at the muscle 6/7 boundary was rarely observed in either *Ugalt* KD1 or *dC1GalT1* KD larvae (Figure 7A–B″), it was frequently observed in the double KD larvae (Figure 7C–C″). We confirmed that the relative level of *dC1GalT1* mRNA in *dC1GalT1* KD larvae was reduced to about 24% of that in control (*Act5C-Gal4/+*) larvae (Figure 7D).

Both the frequency of the Ndg loss phenotype and the total range of Ndg loss at the muscle 6/7 boundary were significantly increased in the double KD larvae as compared with the two single KD larvae (Figure 7E,F). Moreover, the number of NMJ boutons localized just above the site of missing Ndg at the muscle 6/7 boundary was significantly higher in the double KD larvae than in the two single KD larvae (Figure 7G). This number was strongly correlated with the total range of Ndg loss at the muscle 6/7 boundary in the double KD larvae (Figure 7H). Collectively, these data suggest that *Ugalt* and *dC1GalT1* genetically interact with each other.

**Figure 8:**
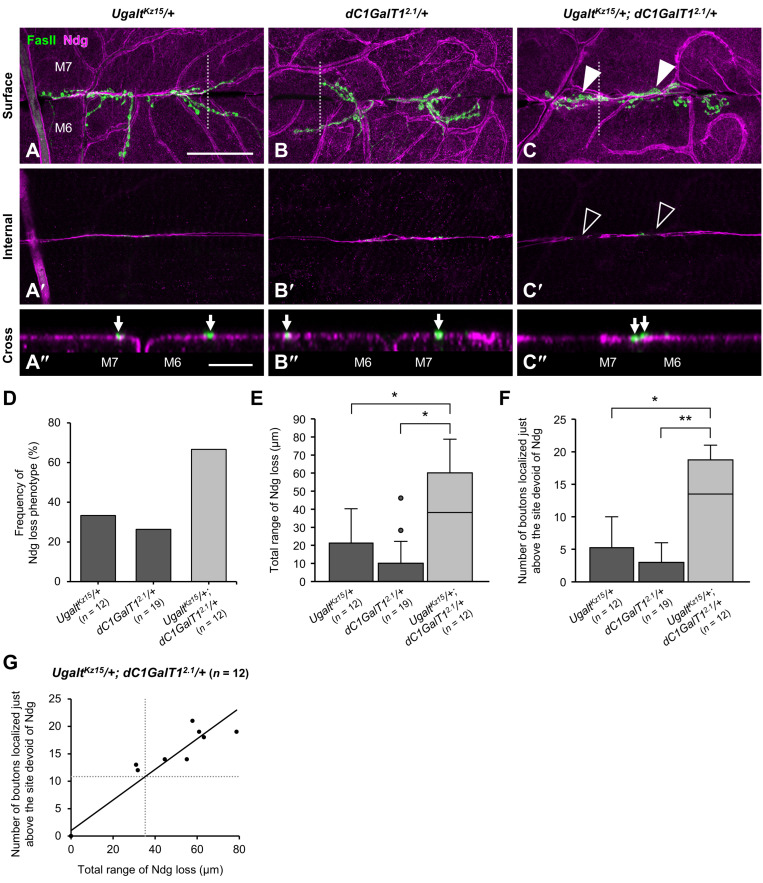
Increase in mislocalized boutons at the muscle 6/7 boundary in double heterozygous mutants of *Ugalt* and *dC1GalT1*. (**A**–**C″**) Confocal images of basement membranes (BMs) and neuromuscular junctions (NMJs) on muscle 6 (M6) and M7 at abdominal segment 3 in *Ugalt^Kz15^* heterozygous (*Ugalt^Kz15^/+*) (**A**–**A″**), *dC1GalT1^2.1^* heterozygous (*dC1GalT1^2.1^/+*) (**B**–**B″**), and *Ugalt^Kz15^* and *dC1GalT1^2.1^* double heterozygous (*Ugalt^Kz15^/+; dC1GalT1^2.1^/+*) (**C**–**C″**) mutant third-instar larvae. Shown are surface (**A**–**C**) and internal (**A′**–**C′**) sectional views of the muscles, and cross-sectional views of areas within the white dotted-lines in (**A**–**C**,**A″**–**C″**). BMs and NMJ boutons were labeled with anti-Ndg antibody (magenta) and anti-Fas II antibody (green), respectively. White arrowheads in (**C**) and black arrowheads in (**C′**) indicate, respectively, mislocalized boutons and partial loss of Ndg staining at the M6/7 boundary. Arrows in (**A″**–**C″**) show positions of NMJ boutons. Scale bars: 50 µm (**A**–**C**,**A′**–**C′**) and 10 µm (**A″**–**C″**). (**D**) Frequency of Ndg loss phenotype in *Ugalt^Kz15^/+* (*n* = 12), *dC1GalT1^2.1^/+* (*n* = 19), and *Ugalt^Kz15^/+; dC1GalT1^2.1^/+* (*n* = 12) larvae. (**E**) Total range of Ndg loss at the M6/7 boundary in *Ugalt^Kz15^/+* (*n* = 12), *dC1GalT1^2.1^/+* (*n* = 19), and *Ugalt^Kz15^/+; dC1GalT1^2.1^/+* (*n* = 12) larvae. (**F**) Number of boutons localized just above the site devoid of Ndg at the M6/7 boundary in *Ugalt^Kz15^/+* (*n* = 12), *dC1GalT1^2.1^/+* (*n* = 19), and *Ugalt^Kz15^/+; dC1GalT1^2.1^/+* (*n* = 12) larvae. In (**E**,**F**), statistical significance was assessed by Steel-Dwass test: * *p* < 0.05, ** *p* < 0.01. (**G**) Correlation between the total range of Ndg loss and the number of boutons localized just above the site devoid of Ndg at the M6/7 boundary in *Ugalt^Kz15^* and *dC1GalT1^2.1^* double heterozygous mutant larvae (*Ugalt^Kz15^/+; dC1GalT1^2.1^/+*) (*n* = 12). Pearson’s correlation coefficient *r* was 0.96. Unbroken line indicates the straight line fit; dotted lines indicate the average values.

To probe the genetic interaction between *Ugalt* and *dC1GalT1* further, we analyzed double heterozygous mutants using *Ugalt^Kz15^* and the null allele *dC1GalT1^2.1^* [60](*Ugalt^Kz15^/+; dC1GalT1^2.1^/+*). Whereas bouton mislocalization accompanied by partial loss of Ndg at the muscle 6/7 boundary was rarely observed in the two single heterozygous mutants (*Ugalt^Kz15^/+* and *dC1GalT1^2.1^/+*) (Figure 8A–B″), it was frequently observed in the double heterozygous mutants (Figure 8C–C″). Furthermore, the frequency of Ndg loss phenotype (Figure 8D), total range of Ndg loss (Figure 8E), and number of boutons localized just above the site of missing Ndg (Figure 8F) were significantly higher in the double heterozygous mutants than in either of the single heterozygous mutants. Moreover, we also found a strong correlation between the range of missing Ndg and the number of mislocalized boutons in the double heterozygous mutants (Figure 8G). Additionally, the PNA blot revealed that the intensities of bands near 75 kDa tended to decrease in the double heterozygous mutants as compared with the two single heterozygous mutants (Supplemental Figures S3 and S5), consistent with the results of the PNA blot in *Ugalt* KD larvae (Figure 1D,E).

**Figure 9:**
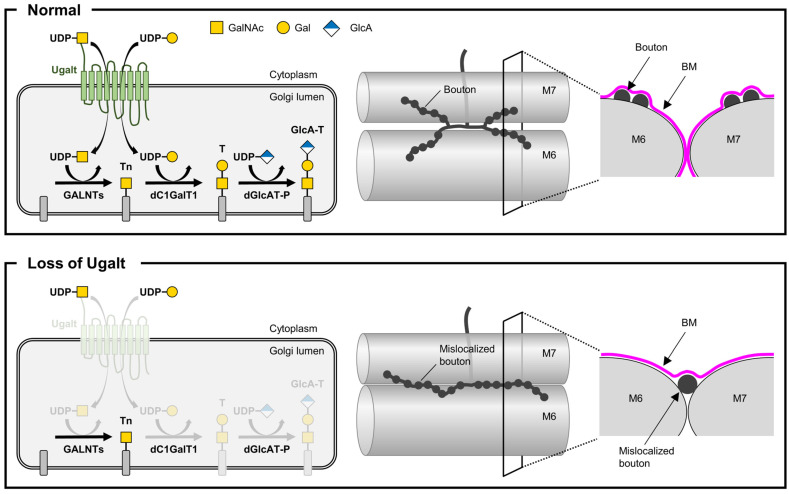
Mechanistic model of the role of Ugalt in the localization of NMJ boutons. **Upper**, Localized in the Golgi membrane, Ugalt facilitates the synthesis of mucin-type *O*-glycans, including Tn antigen, T antigen, and glucuronylated T antigen by transporting uridine diphosphate-*N*-acetylgalactosamine (UDP-GalNAc) and UDP-galactose (UDP-Gal) from the cytoplasm to the Golgi lumen, leading to the precise localization of NMJ boutons and normal formation of basement membrane (BMs) on muscle 6 (M6) and M7. **Lower**, Loss of Ugalt reduces levels of UDP-Gal in the Golgi lumen and causes defective mucin-type *O*-glycosylation, resulting in the mislocalization of NMJ boutons accompanied by the loss of BM components at the M6/7 boundary. Loss of Ugalt also impairs the supply of UDP-GalNAc into the Golgi lumen, which in turn may suppress the increase in Tn antigen levels caused by reduced T antigen synthesis and help to maintain Tn antigen levels. GlcA, glucuronic acid; Tn, Tn antigen; T, T antigen; GlcA-T, glucuronylated T antigen; GALNTs, *N*-acetylgalactosaminyl-transferases; dC1GalT1, *Drosophila* core 1 β1,3-galactosyltransferase 1; dGlcAT-P, *Drosophila* β1,3-glucuronyltransferase-P.

Collectively, these data further demonstrate that *Ugalt* genetically interacts with *dC1GalT1* and that loss of Ugalt causes impaired mucin-type *O*-glycosylation, leading to NMJ bouton mislocalization accompanied by a deficiency of BM components at the muscle 6/7 boundary (Figure 9). In addition, the loss of Ugalt reduces UDP-GalNAc levels in the Golgi lumen, which may suppress the increase in Tn antigen levels caused by reduced T antigen synthesis, thereby maintaining Tn antigen levels within a normal range.

## 4. Discussion

### 4.1. Drosophila Model Unveils the Neurological Basis of SLC35A2-CDG

Patients affected with SLC35A2-CDG have various neurological symptoms, but the underlying pathological mechanism is poorly understood. Here, we have reported the first *Drosophila* model of SLC35A2-CDG, which displays morphological abnormalities of NMJs due to the defective synthesis of mucin-type core 1 glycans. Our findings identify the *Drosophila* ortholog Ugalt as a key factor in mucin-type *O*-glycosylation, which in turn regulates NMJ morphology. Given that *Drosophila* NMJs serve as an excellent model for studies of mammalian central synapses, our findings will provide valuable insights into the links between a specific type of hypogalactosylation and neurological symptoms in SLC35A2-CDG.

### 4.2. Neurological Features in a Newly Identified Case of SLC35A2-CDG

Patients with pathogenic variants in *SLC35A2* (SLC35A2-CDG) are characterized by neurological manifestations, including growth failure, structural brain abnormalities, developmental delay, and epilepsy, as well as other clinical features such as muscle hypotonia, skeletal abnormalities, distinctive facial appearances, and liver dysfunction [72,73]. In our in-house cohort of 900 undiagnosed patients, we identified a male infant carrying a pathogenic variant of *SLC35A2* (c.617_620del, p.(Gln206ArgfsTer45)). Although the presence of epilepsy could not be confirmed due to the patient’s neonatal age, neurological abnormalities such as bilateral ventriculomegaly and reduced head circumference were observed.

### 4.3. Essential Roles of SLC35A2 and Ugalt in Organismal Viability

SLC35A2 is known to have multiple splicing isoforms, including UGT1 and UGT2 [4,5]. Due to differences in the C-terminal amino acid sequence between these two isoforms, UGT1 is localized exclusively to the Golgi, whereas UGT2 is localized to both the ER and Golgi [74]. In contrast, the two splicing isoforms of Ugalt (Ugalt-PA/PC and Ugalt-PB) differ in their N-terminal rather than their C-terminal regions and functional differences between them have not been identified. We found that ubiquitous expression of a gene encoding Ugalt-PA/PC was sufficient to rescue the lethality of *Ugalt* hemizygous mutants, suggesting that Ugalt-PA/PC is essential for viability. Although heterozygous or hemizygous mutations in the X-linked *SLC35A2* gene are known to cause SLC35A2-CDG, most of the affected individuals are females. Males with an *SLC35A2* mutation show genetic mosaicism, indicating that this disorder is X-linked dominant and that a wild-type *SLC35A2* allele is essential for survival [7,75]. Therefore, the functional significance of this transporter during development is common between *Drosophila* and human.

### 4.4. Impact of Ugalt Loss on Mucin-Type O-Glycan Synthesis and Compensation Mechanisms

The present study found that Ugalt is required for the synthesis of mucin-type *O*-glycans, namely, T antigen. Previous studies have shown that SLC35A2-deficient mammalian cells are unable to synthesize mucin-type *O*-glycans [30,31], suggesting that this glycosylation pathway is conserved between mammals and *Drosophila*. Because T antigen is glucuronylated by dGlcAT-P in *Drosophila* [40], loss of Ugalt will also lead to defective synthesis of glucuronylated T antigen. Indeed, our MS analysis revealed reduced levels of glucuronylated T antigen in both *Ugalt* KD1 and KD2 larvae. Moreover, this study revealed that the loss of Ugalt does not affect Tn antigen levels. Given that both human SLC35A2 and Ugalt are known to transport UDP-GalNAc as well as UDP-Gal [6], these data suggest that loss of Ugalt may also reduce Golgi levels of UDP-GalNAc, the substrate essential for Tn antigen synthesis. This reduction in substrate might mitigate the expected increase in Tn antigen levels resulting from impaired T antigen synthesis, thereby maintaining levels within normal range. Conversely, it is plausible that residual Ugalt, which remains due to incomplete suppression by KD, or another UDP-sugar transporter, Fringe connection [76], may supply a small amount of UDP-GalNAc into the Golgi lumen, which in turn would offset any reduction in Tn antigen levels.

### 4.5. Role of Mucin-Type O-Glycans in NMJ Bouton Localization and BM Integrity

Morphological analysis of NMJs revealed that loss of Ugalt did not affect the total bouton number, consistent with our previous analyses in *dC1GalT1* and *dGlcAT-P* mutants [40,62], suggesting that mucin-type *O*-glycans are not involved in bouton addition. However, KD of *Ugalt* did not affect the branch number, whereas the loss of either *dC1GalT1* or *dGlcAT-P* resulted in a reduction in branching [40,62]. These data indicate that residual Ugalt may slightly compensate for the defect in mucin-type *O*-glycosylation, helping to mitigate the phenotype. Our analyses also showed that the loss of Ugalt leads to an increase in the mislocalization of NMJ boutons in association with a deficiency in BM components. This phenotype is identical to that observed in *dC1GalT1* and *dGlcAT-P* mutants [40,41,62]. The finding of a genetic interaction between *Ugalt* and *dC1GalT1* confirms that the bouton mislocalization caused by loss of Ugalt results from defective mucin-type *O*-glycosylation. Depletion of dGlcAT-P is sufficient to induce this phenotype [40]; therefore, glucuronylated T antigen, rather than unmodified T antigen, predominantly coordinates the localization of boutons. Our previous studies supported an association between bouton mislocalization and the loss of BM components [40,41,62]. Given that BMs are formed by components that are secreted from the fat body into the hemolymph and deposited on various tissue surfaces during the larval stages [77], we suggest that the mislocalization of boutons at the muscle 6/7 boundary physically prevents the deposition of BM components just beneath them, leading to the observed partial loss of these components.

### 4.6. Cooperative Roles of Ugalt and dC1GalT1 in T antigen Synthesis and NMJ Morphology

Despite the significant reduction in T antigen levels in both *Ugalt* KD1 and KD2 larvae, only *Ugalt* KD2 larvae exhibited bouton mislocalization and the associated loss of BM components, whereas *Ugalt* KD1 larvae did not display either phenotype. This discrepancy may reflect the higher KD efficiency in KD2 as compared with KD1, as well as the lower T antigen levels in KD2, as demonstrated by MS analysis. These findings suggest that the phenotypes emerge only when the levels of mucin-type *O*-glycans fall below a certain threshold. Further analyses using double KD and double heterozygous mutants confirmed a genetic interaction between *Ugalt* and *dC1GalT1*, likely reflecting their cooperative roles in the biosynthesis of T antigen. Ugalt contributes to the supply of donor substrate UDP-Gal for dC1GalT1, which in turn directly catalyzes the transfer of Gal to synthesize T antigen. When the expression of both genes is partially suppressed, the cumulative reduction in the levels of mucin-type *O*-glycans may fall below a critical threshold, resulting in the emergence of NMJ and BM phenotypes. This suggests that Ugalt and dC1GalT1 function within a shared pathway essential for proper glycosylation and NMJ morphogenesis. SLC35A2 regulates both protein levels and subcellular localization of C1GalT1 and Cosmc through its interactions with these proteins [29]. In *Drosophila*, Ugalt and dC1GalT2, a paralog of dC1GalT1, are localized to the medial cisternae of the Golgi apparatus [21]. These observations raise the possibility of a physical interaction between Ugalt and dC1GalT1 in the Golgi, in addition to their genetic interaction.

### 4.7. Proposed Mechanism of NMJ Bouton Mislocalization via Glycan-Mediated Cell Adhesion

The molecular mechanism underlying bouton mislocalization remains unknown. A previous study showed that overexpression of *Fasciclin I*, encoding a homophilic neural cell adhesion molecule, results in altered NMJ morphologies [78], similar to those observed in *dC1GalT1* and *dGlcAT-P* mutants. This observation indicates that enhanced interactions between neuron and muscle may affect bouton localization. Due to their negative charge, sialylated mucin-type *O*-glycans serve as anti-adhesive molecules in mammals [79,80], negatively charged glucuronylated T antigen may exhibit similar properties in *Drosophila*. We thus propose the idea that reduced glucuronylated T antigens on the cell surface facilitates neuron–muscle interactions, impacting NMJ morphology. Similar phenomena may also occur in the brains of mammals including humans due to defects in the synthesis of mucin-type *O*-glycans.

### 4.8. Linking Synaptic Defects in Drosophila to Brain Abnormalities in SLC35A2-CDG

Our findings provide mechanistic insights into how glycosylation defects may contribute to synaptic abnormalities. Importantly, the *Drosophila* NMJ is a well-established model for studying synaptic development and organization, offering valuable parallels to central synapses in mammals [63]. In patients with SLC35A2-CDG, pathogenic variants of *SLC35A2* cause various neurological symptoms, as mentioned above. These manifestations suggest that glycosylation defects impair neural development and connectivity, potentially through mechanisms analogous to those observed in our *Drosophila* model. Given the evolutionary conservation of glycosylation pathways, it is plausible that similar glycan disruptions in synaptic organization contribute to the neurological phenotypes observed in SLC35A2-CDG patients. Therefore, our findings highlight the utility of the *Drosophila* model in elucidating the cellular and molecular consequences of glycosylation defects and suggest that glycan defects may be a common pathogenic mechanism underlying synaptic and structural brain abnormalities in SLC35A2-CDG.

### 4.9. Potential Role of Mucin-Type O-Glycans in Neurological Symptoms of SLC35A2-CDG

While most patients with SLC35A2-CDG have been shown to display hypogalactosylation of *N*-glycans on serum proteins [7,8,10,11,12,13,14], only a few studies have reported a reduction in mucin-type *O*-glycans [7,10,81]. These data suggest that *N*-glycosylation is more affected than mucin-type *O*-glycosylation on serum proteins in patients with SLC35A2-CDG. However, the impact of SLC35A2 deficiency on these glycosylation pathways in the brain has not been clarified. Mucin-type *O*-glycans are known to account for 70–80% of all *O*-glycans in the mammalian brain [82,83], but their association with human disorders remains largely unknown. Recent studies have identified novel CDGs caused by mutations in *Cosmc* [84] and *GALNT2* [85], both of which play a crucial role in mucin-type *O*-glycosylation, as mentioned above. Patients with X-linked Cosmc-CDG have various symptoms, including developmental delay, intellectual disability, and seizures. Similarly, those with GALNT2-CDG exhibit diverse neurological abnormalities, including intellectual disability, epilepsy, and white matter changes on brain MRI. Thus, mucin-type *O*-glycans have vital functions in the human brain. Given the similarity of these neurological symptoms to those seen in SLC35A2-CDG, and the interaction between Cosmc and SLC35A2 [29], it is plausible that some of the neurological symptoms in SLC35A2-CDG might result from impaired synthesis of mucin-type *O*-glycans in the brain.

### 4.10. A Drosophila CDG Model for Investigating Brain Disorders Caused by O-glycosylation Defects

The hypogalactosylation of *N*-glycans in SLC35A2-CDG is probably due to the reduced substrate for β1,4-galactosyltransferases [86]. Although *Drosophila* has two homologs of human β1,4-galactosyltransferases, these proteins have no Gal transferase activity [87,88]. Galactosylated *N*-glycans are very minor components in *Drosophila*; instead, high mannose and pauci-mannose structures are the major components of glycans [71]. Our MS analyses did not detect galactosylated *N*-glycans and revealed that the loss of Ugalt has minimal impact on overall *N*-glycan levels. By contrast, this study has demonstrated that impaired Ugalt expression leads to reduced mucin-type *O*-glycosylation, causing morphological defects in the model system of mammalian central synapses. We therefore propose that our CDG model serves as a valuable tool to elucidate the causal relationship between defective mucin-type *O*-glycosylation and brain abnormalities. Future studies are needed to clarify the extent to which mucin-type *O*-glycans regulate NMJ morphology, which, in turn, will shed light on the pathological mechanisms underlying SLC35A2-CDG.

## 5. Conclusions

Our analyses have revealed that *Ugalt*, a *Drosophila* ortholog of *SLC35A2*, is required for the synthesis of mucin-type core 1 glycans, which are expressed on the surfaces of muscle and NMJ; *Ugalt* also regulates the localization of NMJ boutons through genetic interaction with *dC1GalT1*. This study has demonstrated that Ugalt is a crucial provider of substrates for mucin-type *O*-glycosylation, which, in turn, coordinates neuromuscular morphology. Our *Drosophila* model will be a valuable model for elucidating the link between impaired mucin-type *O*-glycosylation and the neurological symptoms observed in SLC35A2-CDG.

## Data Availability

The original contributions presented in this study are included in the article/Supplementary Material. Further inquiries can be directed to the corresponding author.

## Supplementary Materials

The following supporting information can be downloaded at: https://www.mdpi.com/article/10.3390/biom15091256/s1, Figure S1: Rescue of the lethality in Ugalt knockout mutants.; Figure S2: Relative whole-lane band intensities in PNA and HPA blots of Ugalt knockdown larvae.; Figure S3: Lectin blot analysis of Ugalt and dC1GalT1 double heterozygous mutants.; Figure S4: Original lectin blot and Western blot images of Figure 1D; Figure S5: Original lectin blot and Western blot images of Figure S3A; Table S1: Primer sets for real-time PCR.; Table S2: Primer sets for first and second PCR.; Video S1: Normal formation of basement membranes at the muscle 6/7 boundary in control larvae.; Video S2: Partial loss of basement membrane components just beneath mislocalized boutons.

## References

[1] Quelhas D., Jaeken J. (2024). Treatment of Congenital Disorders of Glycosylation: An Overview. Mol. Genet. Metab..

[2] Marques-da-Silva D., Francisco R., Webster D., Ferreira V.d.R., Jaeken J., Pulinilkunnil T. (2017). Cardiac Complications of Congenital Disorders of Glycosylation (CDG): A Systematic Review of the Literature. J. Inherit. Metab. Dis..

[3] Paprocka J., Jezela-Stanek A., Tylki-Szymańska A., Grunewald S. (2021). Congenital Disorders of Glycosylation from a Neurological Perspective. Brain Sci..

[4] Miura N., Ishida N., Hoshino M., Yamauchi M., Hara T., Ayusawa D., Kawakita M. (1996). Human UDP-Galactose Translocator: Molecular Cloning of a Complementary DNA That Complements the Genetic Defect of a Mutant Cell Line Deficient in UDP-Galactose Translocator1. J. Biochem..

[5] Ishida N., Miura N., Yoshioka S., Kawakita M. (1996). Molecular Cloning and Characterization of a Novel Isoform of the Human UDP-Galactose Transporter, and of Related Complementary DNAs Belonging to the Nucleotide-Sugar Transporter Gene Family1. J. Biochem..

[6] Segawa H., Kawakita M., Ishida N. (2002). Human and *Drosophila* UDP-galactose Transporters Transport UDP-*N*-acetylgalactosamine in Addition to UDP-galactose. Eur. J. Biochem..

[7] Ng B.G., Buckingham K.J., Raymond K., Kircher M., Turner E.H., He M., Smith J.D., Eroshkin A., Szybowska M., Losfeld M.E. (2013). Mosaicism of the UDP-Galactose Transporter *SLC35A2* Causes a Congenital Disorder of Glycosylation. Am. J. Hum. Genet..

[8] Xia B., Zhang W., Li X., Jiang R., Harper T., Liu R., Cummings R.D., He M. (2013). Serum N-Glycan and O-Glycan Analysis by Mass Spectrometry for Diagnosis of Congenital Disorders of Glycosylation. Anal. Biochem..

[9] Kodera H., Nakamura K., Osaka H., Maegaki Y., Haginoya K., Mizumoto S., Kato M., Okamoto N., Iai M., Kondo Y. (2013). De Novo Mutations in *SLC35A2* Encoding a UDP-Galactose Transporter Cause Early-Onset Epileptic Encephalopathy. Hum. Mutat..

[10] Bruneel A., Cholet S., Drouin-Garraud V., Jacquemont M., Cano A., Mégarbané A., Ruel C., Cheillan D., Dupré T., Vuillaumier-Barrot S. (2018). Complementarity of Electrophoretic, Mass Spectrometric, and Gene Sequencing Techniques for the Diagnosis and Characterization of Congenital Disorders of Glycosylation. Electrophoresis.

[11] Vals M., Ashikov A., Ilves P., Loorits D., Zeng Q., Barone R., Huijben K., Sykut-Cegielska J., Diogo L., Elias A.F. (2019). Clinical, Neuroradiological, and Biochemical Features of SLC35A2-CDG Patients. J. Inherit. Metab. Dis..

[12] Witters P., Tahata S., Barone R., Õunap K., Salvarinova R., Grønborg S., Hoganson G., Scaglia F., Lewis A.M., Mori M. (2020). Clinical and Biochemical Improvement with Galactose Supplementation in SLC35A2-CDG. Genet. Med..

[13] Wada Y. (2020). Matrix-Assisted Laser Desorption/Ionization Mass Spectrometry to Detect Diagnostic Glycopeptide Markers of Congenital Disorders of Glycosylation. Mass. Spectrom..

[14] Kodríková R., Pakanová Z., Krchňák M., Šedivá M., Šesták S., Květoň F., Beke G., Šalingová A., Skalická K., Brennerová K. (2023). *N*-Glycoprofiling of SLC35A2-CDG: Patient with a Novel Hemizygous Variant. Biomedicines.

[15] Bonduelle T., Hartlieb T., Baldassari S., Sim N.S., Kim S.H., Kang H.-C., Kobow K., Coras R., Chipaux M., Dorfmüller G. (2021). Frequent *SLC35A2* Brain Mosaicism in Mild Malformation of Cortical Development with Oligodendroglial Hyperplasia in Epilepsy (MOGHE). Acta Neuropathol. Commun..

[16] Barba C., Blumcke I., Winawer M.R., Hartlieb T., Kang H.-C., Grisotto L., Chipaux M., Bien C.G., Heřmanovská B., Porter B.E. (2023). Clinical Features, Neuropathology, and Surgical Outcome in Patients With Refractory Epilepsy and Brain Somatic Variants in the *SLC35A2* Gene. Neurology.

[17] Elziny S., Sran S., Yoon H., Corrigan R.R., Page J., Ringland A., Lanier A., Lapidus S., Foreman J., Heinzen E.L. (2024). Loss of *Slc35a2* Alters Development of the Mouse Cerebral Cortex. Neurosci. Lett..

[18] Spyrou J., Aung K.P., Vanyai H., Leventer R.J., Maljevic S., Lockhart P.J., Howell K.B., Reid C.A. (2024). *Slc35a2* Mosaic Knockout Impacts Cortical Development, Dendritic Arborisation, and Neuronal Firing. Neurobiol. Dis..

[19] Yoon H., Ringland A., Anderson J.J., Sran S., Elziny S., Huynh C., Shinagawa N., Badertscher S., Corrigan R.R., Mashburn-Warren L. (2024). Mouse Models of *Slc35a2* Brain Mosaicism Reveal Mechanisms of Mild Malformations of Cortical Development with Oligodendroglial Hyperplasia in Epilepsy. Epilepsia.

[20] Aumiller J.J., Jarvis D.L. (2002). Expression and Functional Characterization of a Nucleotide Sugar Transporter from *Drosophila Melanogaster*: Relevance to Protein Glycosylation in Insect Cell Expression Systems. Protein Expr. Purif..

[21] Yamamoto-Hino M., Abe M., Shibano T., Setoguchi Y., Awano W., Ueda R., Okano H., Goto S. (2012). Cisterna-Specific Localization of Glycosylation-Related Proteins to the Golgi Apparatus. Cell Struct. Funct..

[22] Yamamoto-Hino M., Yoshida H., Ichimiya T., Sakamura S., Maeda M., Kimura Y., Sasaki N., Aoki-Kinoshita K.F., Kinoshita-Toyoda A., Toyoda H. (2015). Phenotype-based Clustering of Glycosylation-related Genes by RNAi-mediated Gene Silencing. Genes. Cells.

[23] Maszczak-Seneczko D., Sosicka P., Kaczmarek B., Majkowski M., Luzarowski M., Olczak T., Olczak M. (2015). UDP-Galactose (SLC35A2) and UDP-N-Acetylglucosamine (SLC35A3) Transporters Form Glycosylation-Related Complexes with Mannoside Acetylglucosaminyltransferases (Mgats). J. Biol. Chem..

[24] Khoder-Agha F., Sosicka P., Conde M.E., Hassinen A., Glumoff T., Olczak M., Kellokumpu S. (2019). *N*-Acetylglucosaminyltransferases and Nucleotide Sugar Transporters Form Multi-Enzyme–Multi-Transporter Assemblies in Golgi Membranes In Vivo. Cell Mol. Life Sci..

[25] Wiertelak W., Sosicka P., Olczak M., Maszczak-Seneczko D. (2020). Analysis of Homologous and Heterologous Interactions between UDP-Galactose Transporter and Beta-1,4-Galactosyltransferase 1 Using NanoBiT. Anal. Biochem..

[26] Wiertelak W., Pavlovskyi A., Olczak M., Maszczak-Seneczko D. (2025). Cytosolic UDP-Gal Biosynthetic Machinery Is Required for Dimerization of SLC35A2 in the Golgi Membrane and Its Interaction with B4GalT1. Front. Mol. Biosci..

[27] Shauchuk A., Szulc B., Maszczak-Seneczko D., Wiertelak W., Skurska E., Olczak M. (2020). N-Glycosylation of the Human Β1,4-Galactosyltransferase 4 Is Crucial for Its Activity and Golgi Localization. Glycoconj. J..

[28] Sprong H., Degroote S., Nilsson T., Kawakita M., Ishida N., van der Sluijs P., van Meer G. (2003). Association of the Golgi UDP-Galactose Transporter with UDP-Galactose:Ceramide Galactosyltransferase Allows UDP-Galactose Import in the Endoplasmic Reticulum. Mol. Biol. Cell.

[29] Wiertelak W., Chabowska K., Szulc B., Zadorozhna Y., Olczak M., Maszczak-Seneczko D. (2023). SLC35A2 Deficiency Reduces Protein Levels of Core 1 β-1,3-Galactosyltransferase 1 (C1GalT1) and Its Chaperone Cosmc and Affects Their Subcellular Localization. Biochim. Biophys. Acta (BBA)—Mol. Cell Res..

[30] Påhlsson P., Blackall D.P., Ugorski M., Czerwinski M., Spitalnik S.L. (1994). Biochemical Characterization of The *O*-Glycans on Recombinant Glycophorin A Expressed in Chinese Hamster Ovary Cells. Glycoconj. J..

[31] Szulc B., Sosicka P., Maszczak-Seneczko D., Skurska E., Shauchuk A., Olczak T., Freeze H.H., Olczak M. (2020). Biosynthesis of GlcNAc-Rich *N*- and *O*-Glycans in the Golgi Apparatus Does Not Require the Nucleotide Sugar Transporter SLC35A3. J. Biol. Chem..

[32] Bennett E.P., Mandel U., Clausen H., Gerken T.A., Fritz T.A., Tabak L.A. (2012). Control of Mucin-Type O-Glycosylation: A Classification of the Polypeptide GalNAc-Transferase Gene Family. Glycobiology.

[33] Tran D.T., Hagen K.G. (2013). Mucin-Type *O*-Glycosylation during Development. J. Biol. Chem..

[34] Ju T., Cummings R.D. (2002). A Unique Molecular Chaperone Cosmc Required for Activity of the Mammalian Core 1 β3-Galactosyltransferase. Proc. Natl. Acad. Sci. USA.

[35] Müller R., Hülsmeier A.J., Altmann F., Hagen K.T., Tiemeyer M., Hennet T. (2005). Characterization of Mucin-Type Core-1 β1-3 Galactosyltransferase Homologous Enzymes in *Drosophila Melanogaster*. FEBS J..

[36] Kim B.-T.T., Tsuchida K., Lincecum J., Kitagawa H., Bernfield M., Sugahara K. (2003). Identification and Characterization of Three *Drosophila Melanogaster* Glucuronyltransferases Responsible for the Synthesis of the Conserved Glycosaminoglycan-Protein Linkage Region of Proteoglycans. Two Novel Homologs Exhibit Broad Specificity toward Oligosaccharides from Proteoglycans, Glycoproteins, and Glycosphingolipids. J. Biol. Chem..

[37] Aoki K., Porterfield M., Lee S.S., Dong B., Nguyen K., McGlamry K.H., Tiemeyer M. (2008). The Diversity of *O*-Linked Glycans Expressed during *Drosophila Melanogaster* Development Reflects Stage- and Tissue-Specific Requirements for Cell Signaling. J. Biol. Chem..

[38] Breloy I., Schwientek T., Lehr S., Hanisch F.-G.G. (2008). Glucuronic Acid Can Extend O-Linked Core 1 Glycans, but It Contributes Only Weakly to the Negative Surface Charge of *Drosophila Melanogaster* Schneider-2 Cells. FEBS Lett..

[39] Breloy I., Schwientek T., Althoff D., Holz M., Koppen T., Krupa A., Hanisch F.-G. (2016). Functional Analysis of the Glucuronyltransferases GlcAT-P and GlcAT-S of *Drosophila Melanogaster*: Distinct Activities towards the O-Linked T-Antigen. Biomolecules.

[40] Itoh K., Akimoto Y., Kondo S., Ichimiya T., Aoki K., Tiemeyer M., Nishihara S. (2018). Glucuronylated Core 1 Glycans Are Required for Precise Localization of Neuromuscular Junctions and Normal Formation of Basement Membranes on *Drosophila* Muscles. Dev. Biol..

[41] Itoh K., Nishihara S. (2021). Mucin-Type *O*-Glycosylation in the *Drosophila* Nervous System. Front. Neuroanat..

[42] Berger E.G. (1999). Tn-Syndrome. Biochim. Biophys. Acta.

[43] Ju T., Cummings R.D. (2005). Protein Glycosylation: Chaperone Mutation in Tn Syndrome. Nature.

[44] Suzuki H., Moldoveanu Z., Hall S., Brown R., Vu H.L., Novak L., Julian B.A., Tomana M., Wyatt R.J., Edberg J.C. (2008). IgA1-Secreting Cell Lines from Patients with IgA Nephropathy Produce Aberrantly Glycosylated IgA1. J. Clin. Investig..

[45] Hiki Y. (2009). *O*-Linked Oligosaccharides of the IgA1 Hinge Region: Roles of Its Aberrant Structure in the Occurrence and/or Progression of IgA Nephropathy. Clin. Exp. Nephrol..

[46] Springer G. (1984). T and Tn, General Carcinoma Autoantigens. Science.

[47] Ju T., Lanneau G.S., Gautam T., Wang Y., Xia B., Stowell S.R., Willard M.T., Wang W., Xia J.Y., Zuna R.E. (2008). Human Tumor Antigens Tn and Sialyl Tn Arise from Mutations in *Cosmc*. Cancer Res..

[48] Radhakrishnan P., Dabelsteen S., Madsen F.B., Francavilla C., Kopp K.L., Steentoft C., Vakhrushev S.Y., Olsen J.V., Hansen L., Bennett E.P. (2014). Immature Truncated O-Glycophenotype of Cancer Directly Induces Oncogenic Features. Proc. Natl. Acad. Sci. USA.

[49] Xia L., Ju T., Westmuckett A., An G., Ivanciu L., McDaniel J.M., Lupu F., Cummings R.D., McEver R.P. (2004). Defective Angiogenesis and Fatal Embryonic Hemorrhage in Mice Lacking Core 1-Derived O-Glycans. J. Cell Biol..

[50] Wang Y., Jobe S.M., Ding X., Choo H., Archer D.R., Mi R., Ju T., Cummings R.D. (2012). Platelet Biogenesis and Functions Require Correct Protein O-Glycosylation. Proc. Natl. Acad. Sci. USA.

[51] Kudo T., Sato T., Hagiwara K., Kozuma Y., Yamaguchi T., Ikehara Y., Hamada M., Matsumoto K., Ema M., Murata S. (2013). C1galt1-Deficient Mice Exhibit Thrombocytopenia Due to Abnormal Terminal Differentiation of Megakaryocytes. Blood.

[52] Fu J., Wei B., Wen T., Johansson M.E., Liu X., Bradford E., Thomsson K.A., McGee S., Mansour L., Tong M. (2011). Loss of Intestinal Core 1-Derived *O*-Glycans Causes Spontaneous Colitis in Mice. J. Clin. Investig..

[53] Bergstrom K., Liu X., Zhao Y., Gao N., Wu Q., Song K., Cui Y., Li Y., McDaniel J.M., McGee S. (2016). Defective Intestinal Mucin-Type *O*-Glycosylation Causes Spontaneous Colitis-Associated Cancer in Mice. Gastroenterology.

[54] Fuseya S., Suzuki R., Okada R., Hagiwara K., Sato T., Narimatsu H., Yokoi H., Kasahara M., Usui T., Morito N. (2020). Mice Lacking Core 1-Derived *O*-Glycan in Podocytes Develop Transient Proteinuria, Resulting in Focal Segmental Glomerulosclerosis. Biochem. Biophys. Res. Commun..

[55] Stotter B.R., Talbot B.E., Capen D.E., Artelt N., Zeng J., Matsumoto Y., Endlich N., Cummings R.D., Schlondorff J.S. (2020). Cosmc-Dependent Mucin-Type *O*-Linked Glycosylation Is Essential for Podocyte Function. Am. J. Physiol.-Ren..

[56] Noel M., Suttapitugsakul S., Cummings R.D., Mealer R.G. (2025). O-GalNAc Glycans Are Enriched in Neuronal Tracts and Regulate Nodes of Ranvier. Proc. Natl. Acad. Sci. USA.

[57] Valoskova K., Biebl J., Roblek M., Emtenani S., Gyoergy A., Misova M., Ratheesh A., Reis-Rodrigues P., Shkarina K., Larsen I. (2019). A Conserved Major Facilitator Superfamily Member Orchestrates a Subset of O-Glycosylation to Aid Macrophage Tissue Invasion. eLife.

[58] Yoshida H., Fuwa T.J., Arima M., Hamamoto H., Sasaki N., Ichimiya T., Osawa K., Ueda R., Nishihara S. (2008). Identification of the *Drosophila Core 1 β1,3-Galactosyltransferase* Gene That Synthesizes T Antigen in the Embryonic Central Nervous System and Hemocytes. Glycobiology.

[59] Fuwa T.J., Kinoshita T., Nishida H., Nishihara S. (2015). Reduction of T Antigen Causes Loss of Hematopoietic Progenitors in *Drosophila* through the Inhibition of Filopodial Extensions from the Hematopoietic Niche. Dev. Biol..

[60] Lin Y., Reddy B.V.V.G., Irvine K.D. (2008). Requirement for a Core 1 Galactosyltransferase in the *Drosophila* Nervous System. Dev. Dyn..

[61] Lacin H., Zhu Y., DiPaola J.T., Wilson B.A., Zhu Y., Skeath J.B. (2024). A Genetic Screen in *Drosophila* Uncovers a Role for *Senseless-2* in Surface Glia in the Peripheral Nervous System to Regulate CNS Morphology. G3 Genes Genomes Genet..

[62] Itoh K., Akimoto Y., Fuwa T.J., Sato C., Komatsu A., Nishihara S. (2016). Mucin-Type Core 1 Glycans Regulate the Localization of Neuromuscular Junctions and Establishment of Muscle Cell Architecture in *Drosophila*. Dev. Biol..

[63] Menon K.P., Carrillo R.A., Zinn K. (2013). Development and Plasticity of the *Drosophila* Larval Neuromuscular Junction. Wiley Interdiscip. Rev. Dev. Biol..

[64] Aoki E., Manabe N., Ohno S., Aoki T., Furukawa J.-I., Togayachi A., Aoki-Kinoshita K., Inokuchi J.-I., Kurosawa K., Kaname T. (2023). Predicting the Pathogenicity of Missense Variants Based on Protein Instability to Support Diagnosis of Patients with Novel Variants of ARSL. Mol. Genet. Metab. Rep..

[65] Ohno S., Ogura C., Yabuki A., Itoh K., Manabe N., Angata K., Togayachi A., Aoki-Kinoshita K., Furukawa J., Inamori K. (2025). VarMeter2: An Enhanced Structure-Based Methodfmovi for Predicting Pathogenic Missense Variants through Mahalanobis Distance. Comput. Struct. Biotechnol. J..

[66] Kondo S., Ueda R. (2013). Highly Improved Gene Targeting by Germline-Specific Cas9 Expression in *Drosophila*. Genetics.

[67] Hanamatsu H., Yokota I., Kurogochi M., Akasaka-Manya K., Miura N., Manya H., Endo T., Nishikaze T., Furukawa J., Tanaka K. (2024). Direct Derivatization of Sialic Acids and Mild β-Elimination for Linkage-Specific Sialyl *O*-Glycan Analysis. Anal. Chim. Acta.

[68] Furukawa J., Shinohara Y., Kuramoto H., Miura Y., Shimaoka H., Kurogochi M., Nakano M., Nishimura S.-I. (2008). Comprehensive Approach to Structural and Functional Glycomics Based on Chemoselective Glycoblotting and Sequential Tag Conversion. Anal. Chem..

[69] Hanamatsu H., Nishikaze T., Miura N., Piao J., Okada K., Sekiya S., Iwamoto S., Sakamoto N., Tanaka K., Furukawa J. (2018). Sialic Acid Linkage Specific Derivatization of Glycosphingolipid Glycans by Ring-Opening Aminolysis of Lactones. Anal. Chem..

[70] Ueyama M., Akimoto Y., Ichimiya T., Ueda R., Kawakami H., Aigaki T., Nishihara S. (2010). Increased Apoptosis of Myoblasts in *Drosophila* Model for the Walker-Warburg Syndrome. PLoS ONE.

[71] Aoki K., Perlman M., Lim J.-M., Cantu R., Wells L., Tiemeyer M. (2007). Dynamic Developmental Elaboration of *N*-Linked Glycan Complexity in the *Drosophila Melanogaster* Embryo. J. Biol. Chem..

[72] Quelhas D., Correia J., Jaeken J., Azevedo L., Lopes-Marques M., Bandeira A., Keldermans L., Matthijs G., Sturiale L., Martins E. (2021). SLC35A2-CDG: Novel Variant and Review. Mol. Genet. Metab. Rep..

[73] Yüksel M.F., Doğulu N., Yıldırım M., Köse E., Bektaş Ö., Eminoğlu F.T., Teber S. (2024). Metabolic Etiologies in Children with Infantile Epileptic Spasm Syndrome: Experience at a Tertiary Pediatric Neurology Center. Brain Dev..

[74] Kabuß R., Ashikov A., Oelmann S., Gerardy-Schahn R., Bakker H. (2005). Endoplasmic Reticulum Retention of the Large Splice Variant of the UDP-Galactose Transporter Is Caused by a Dilysine Motif. Glycobiology.

[75] Westenfield K., Sarafoglou K., Speltz L.C., Pierpont E.I., Steyermark J., Nascene D., Bower M., Pierpont M.E. (2018). Mosaicism of the UDP-Galactose Transporter *SLC35A2* in a Female Causing a Congenital Disorder of Glycosylation: A Case Report. BMC Med. Genet..

[76] Goto S., Taniguchi M., Muraoka M., Toyoda H., Sado Y., Kawakita M., Hayashi S. (2001). UDP–Sugar Transporter Implicated in Glycosylation and Processing of Notch. Nat. Cell Biol..

[77] Pastor-Pareja J.C., Xu T. (2011). Shaping Cells and Organs in *Drosophila* by Opposing Roles of Fat Body-Secreted Collagen IV and Perlecan. Dev. Cell.

[78] Zhong Y., Shanley J. (1995). Altered Nerve Terminal Arborization and Synaptic Transmission in *Drosophila* Mutants of Cell Adhesion Molecule Fasciclin I. J. Neurosci..

[79] Takeda T., Go W., Orlando R., Farquhar M. (2000). Expression of Podocalyxin Inhibits Cell-Cell Adhesion and Modifies Junctional Properties in Madin-Darby Canine Kidney Cells. Mol. Biol. Cell.

[80] Doyonnas R., Kershaw D.B., Duhme C., Merkens H., Chelliah S., Graf T., McNagny K.M. (2001). Anuria, Omphalocele, and Perinatal Lethality in Mice Lacking the CD34-Related Protein Podocalyxin. J. Exp. Med..

[81] Wada Y., Okamoto N. (2020). Apolipoprotein C-III *O*-glycoform Profiling of 500 Serum Samples by Matrix-Assisted Laser Desorption/Ionization Mass Spectrometry for Diagnosis of Congenital Disorders of Glycosylation. J. Mass. Spectrom..

[82] Wilkinson H., Thomsson K.A., Rebelo A.L., Hilliard M., Pandit A., Rudd P.M., Karlsson N.G., Saldova R. (2021). The *O*-Glycome of Human Nigrostriatal Tissue and Its Alteration in Parkinson’s Disease. J. Proteome Res..

[83] Williams S.E., Noel M., Lehoux S., Cetinbas M., Xavier R.J., Sadreyev R.I., Scolnick E.M., Smoller J.W., Cummings R.D., Mealer R.G. (2022). Mammalian Brain Glycoproteins Exhibit Diminished Glycan Complexity Compared to Other Tissues. Nat. Commun..

[84] Erger F., Aryal R.P., Reusch B., Matsumoto Y., Meyer R., Zeng J., Knopp C., Noel M., Muerner L., Wenzel A. (2023). Germline *C1GALT1C1* Mutation Causes a Multisystem Chaperonopathy. Proc. Natl. Acad. Sci. USA.

[85] Zilmer M., Edmondson A.C., Khetarpal S.A., Alesi V., Zaki M.S., Rostasy K., Madsen C.G., Lepri F.R., Sinibaldi L., Cusmai R. (2020). Novel Congenital Disorder of *O*-Linked Glycosylation Caused by GALNT2 Loss of Function. Brain.

[86] Ng B.G., Sosicka P., Agadi S., Almannai M., Bacino C.A., Barone R., Botto L.D., Burton J.E., Carlston C., Chung B.H.-Y. (2019). SLC35A2-CDG: Functional Characterization, Expanded Molecular, Clinical, and Biochemical Phenotypes of 30 Unreported Individuals. Hum. Mutat..

[87] Haines N., Irvine K.D. (2005). Functional Analysis of *Drosophila* β1,4-N-Acetlygalactosaminyltransferases. Glycobiology.

[88] Sasaki N., Yoshida H., Fuwa T.J., Kinoshita-Toyoda A., Toyoda H., Hirabayashi Y., Ishida H., Ueda R., Nishihara S. (2006). *Drosophila* β1,4-*N*-Acetylgalactosaminyltransferase-A Synthesizes the LacdiNAc Structures on Several Glycoproteins and Glycosphingolipids. Biochem. Biophys. Res. Commun..

