# Supplementary material for: Neuromuscular Defects in a Drosophila Model of the Congenital Disorder of Glycosylation SLC35A2-CDG
Source: Biomolecules. 2025 Aug 29;15(9):1256. doi: 10.3390/biom15091256 (PMC12467441; doi:10.3390/biom15091256)
Supplement: Supplementary file 1 [file biomolecules-15-01256-s001.zip › biomolecules-3725434-supplementary.pdf]

Supplemental materials

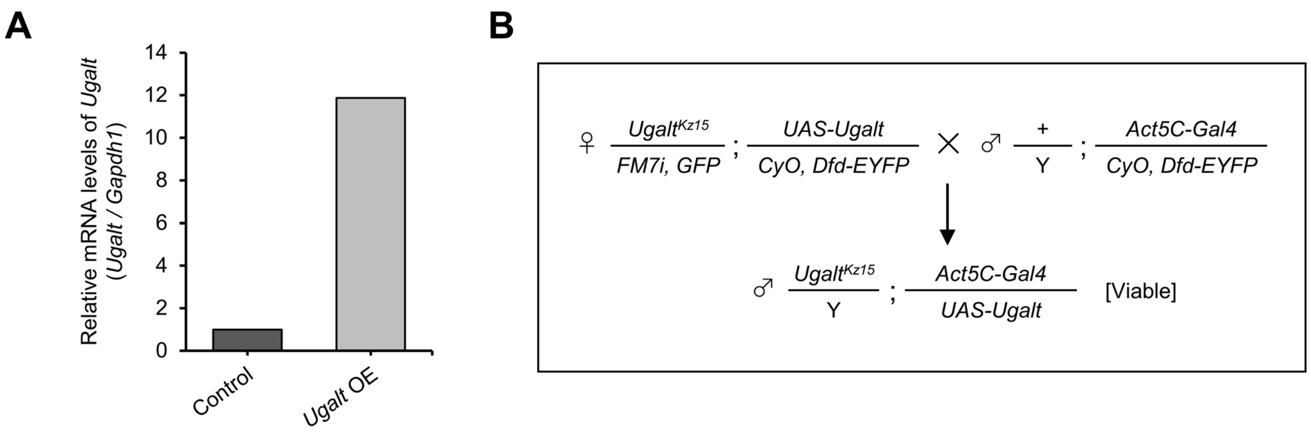

Supplemental Figure S1. Rescue of the lethality in *Ugalt* knockout mutants.

(A) Relative mRNA levels of *Ugalt* in *Ugalt*-overexpressing larvae using *Act5C-Gal4*. The mRNA level of *Ugalt* in the control (*Act5C-Gal4*/+) larvae was set as 1.0.

(B) Crossing scheme for the rescue of *Ugalt*^{Kz15} hemizygous (*Ugalt*^{Kz15}/Y) flies. Female *UAS-Ugalt* flies with *Ugalt*^{Kz15} allele were crossed with male *Act5C-Gal4* driver flies.

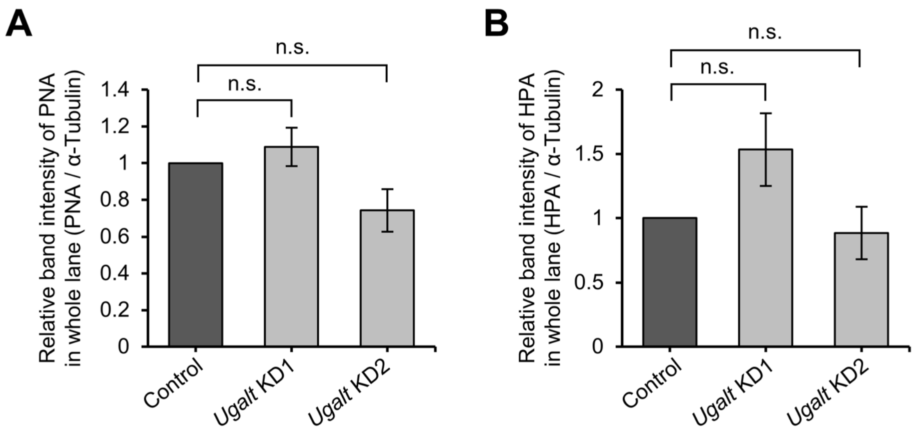

Supplemental Figure S2. Relative whole-lane band intensities in PNA and HPA blots of *Ugalt* knockdown larvae.

Relative whole-lane band intensities of PNA **(A)** and HPA **(B)** in the blots in Fig. 1D, normalized to the band intensity of α-tubulin. Data are the mean ± standard error for each genotype (n = 3). Statistical significance was assessed by Dunnett test: n.s., not significant.

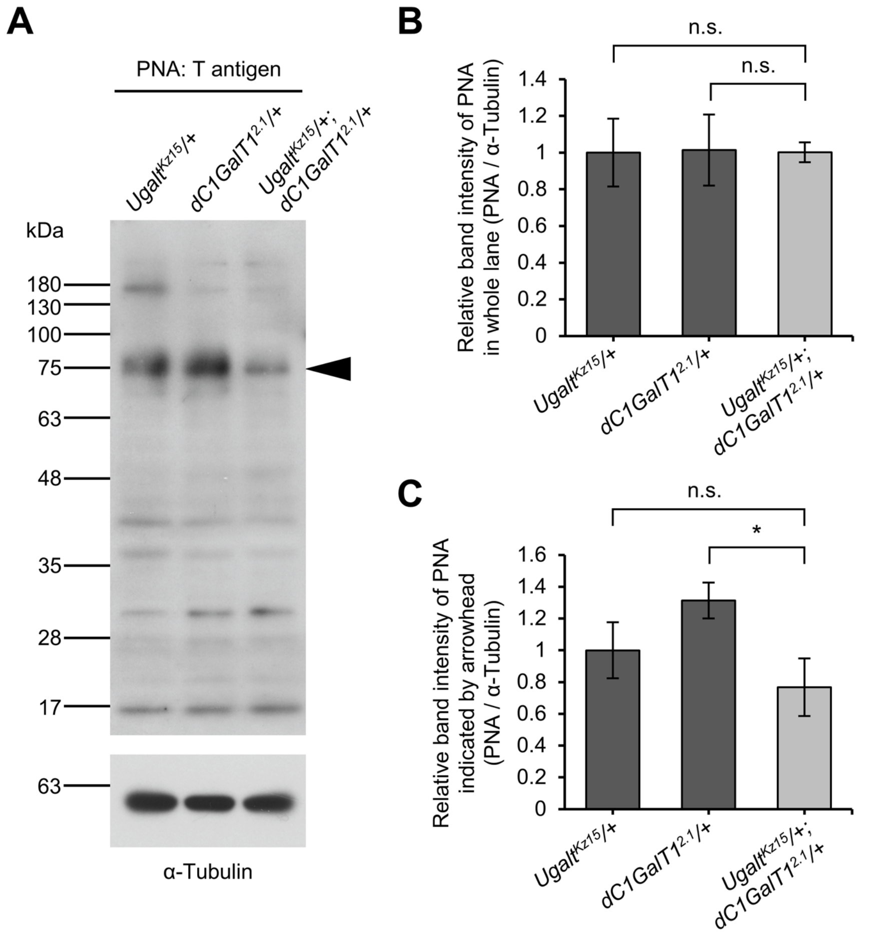

Supplemental Figure S3. Lectin blot analysis of *Ugalt* and *dC1GalT1* double heterozygous mutants.

(A) PNA lectin blot analysis of proteins extracted from the body walls in $Ugalt^{Kz15}$ heterozygous ($Ugalt^{Kz15}$/+), $dC1GalT1^{2.1}$ heterozygous ($dC1GalT1^{2.1}$/+), and $Ugalt^{Kz15}$ and $dC1GalT1^{2.1}$ double heterozygous ($Ugalt^{Kz15}$/+; $dC1GalT1^{2.1}$/+) mutant third-instar larvae. The α-tubulin internal control is shown below. A representative example of three experiments is shown.

(B and C) Relative band intensities of PNA in whole lane (B) and in the band indicated by arrowhead in A (C), normalized to the band intensity of α-tubulin. Data are the mean ± standard error for each genotype (n = 3). Statistical significance was assessed by Dunnett test: $^*p < 0.05$, n.s., not significant. Original lectin blot and Western blot images can be found in Figure S5.

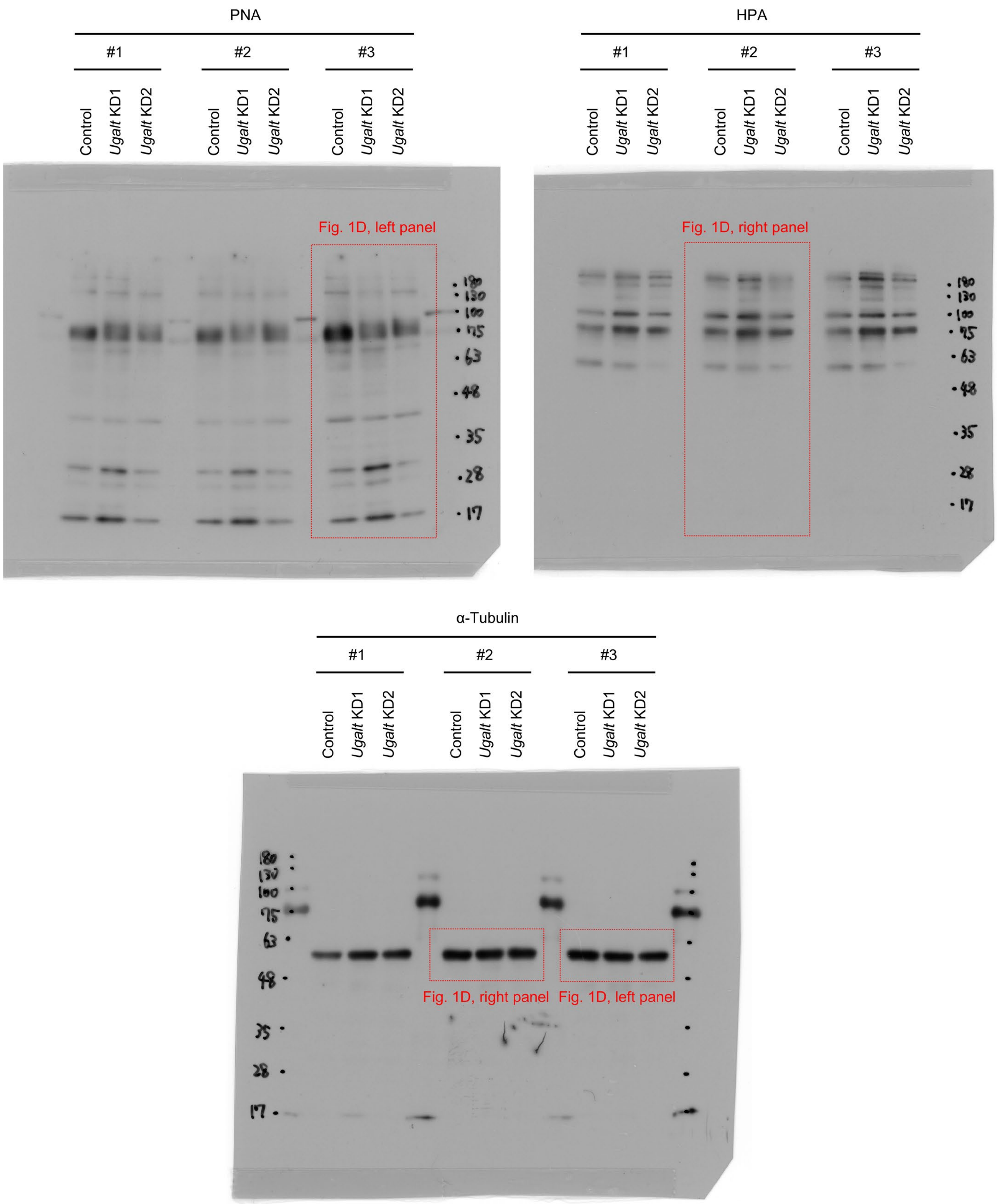

Supplemental Figure S4. Original lectin blot and Western blot images of Figure 1D.

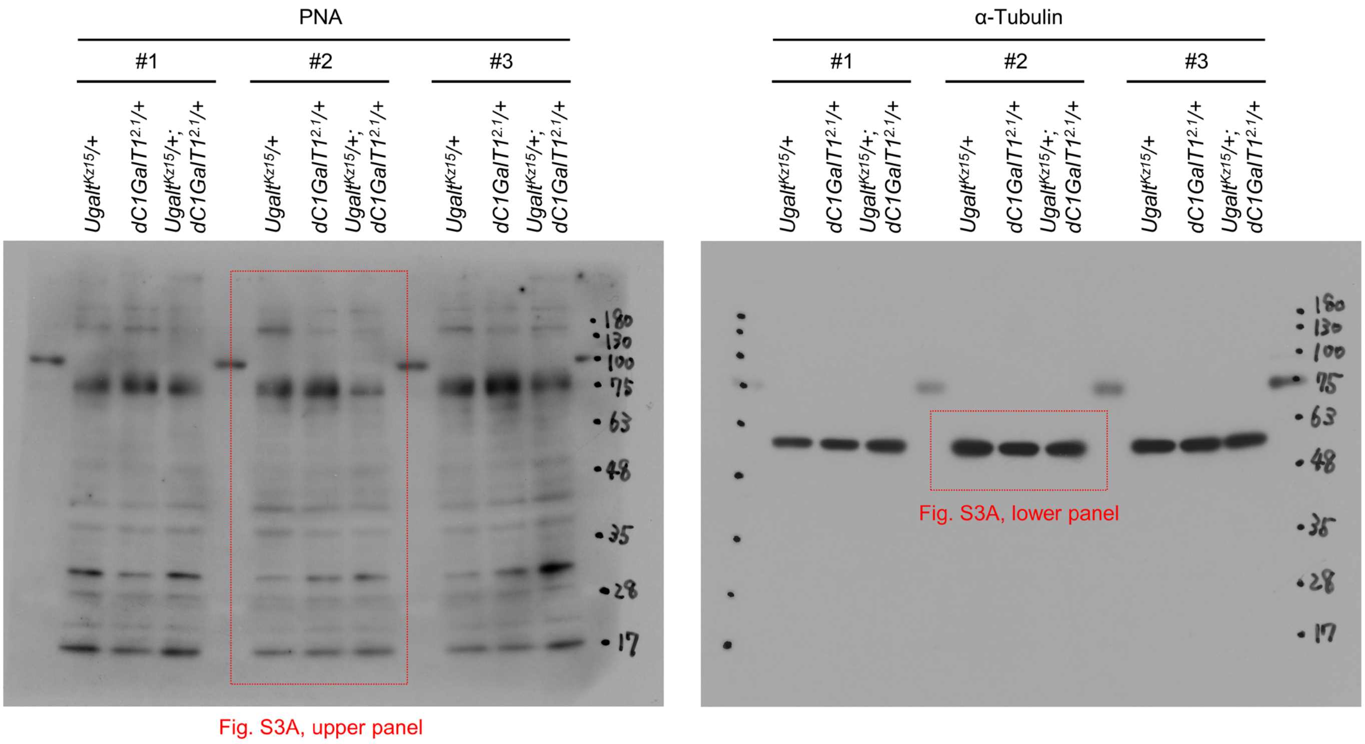

Supplemental Figure S5. Original lectin blot and Western blot images of Figure S3A.

Supplemental Table S1. Primer sets for real-time PCR.

Gene	Forward primer (5' > 3')	Reverse primer (5' > 3')
RpL32	GCAAGCCCAAGGGTATCGA	CGATGTTGGGCATCAGATACTG
Gapdh1	TAAATTCGACTCGACTCACGGT	CTCCACCACATACTCGGCTC
Ugalt	TGAACGCCAATACGCTGAAG	GGCGAACTCTGCCATGAGTAC
dC1GalT1	AAGCCAAATGACTGCCAACAG	ACGATCAGGCCCACAATCAG

Supplemental Table S2. Primer sets for first and second PCR.

1st/2nd PCR	Target gene	Forward primer (5' > 3')	Reverse primer (5' > 3')
1st PCR	*Ugalt*	AAAAAGCAGGCTCAAAATGA<br>ATAGCATACACATGAACGCCA	AGAAAGCTGGGTCCTAGACG<br>CGCGGCAGTAGC
2nd PCR	-	GGGGACAAGTTTGTACAAAAA<br>AGCAGGCT	GGGGACCACTTTGTACAAGAA<br>AGCTGGGT

Supplemental Video S1. Normal formation of basement membranes at the muscle 6/7 boundary in control larvae.
Surface rendering models of three-dimensional confocal images of basement membranes (BMs) and neuromuscular junctions (NMJs) in control (*Act5C-Gal4*/+) larvae. BMs, NMJ boutons, and muscle fibers were labeled with anti-Ndg antibody (magenta), anti-Fas II antibody (green), and phalloidin (white), respectively.

Supplemental Video S2. Partial loss of basement membrane components just beneath mislocalized boutons.
Surface rendering models of three-dimensional confocal images of basement membranes (BMs) and neuromuscular junctions (NMJs) in *Ugalt* KD2 larvae. BMs, NMJ boutons, and muscle fibers were labeled with anti-Ndg antibody (magenta), anti-Fas II antibody (green), and phalloidin (white), respectively.
